# Modulation of Oncogenic NOTCH Signaling in Highly Aggressive Malignancies by Targeting the γ-Secretase Complex: A Systematic Review

**DOI:** 10.3390/cells15050468

**Published:** 2026-03-05

**Authors:** Pablo Martínez-Gascueña, María-Luisa Nueda, Victoriano Baladrón

**Affiliations:** 1 Servicio de Cardiología, Hospital Clínico San Carlos, C/del Profesor Martín Lagos, S/N, Moncloa-Aravaca, 29040 Madrid, Spain; pgascu@outlook.es; 2 Área de Bioquímica y Biología Molecular, Departamento de Química Inorgánica, Orgánica y Bioquímica, Facultad de Farmacia/IB-UCLM/Unidad de Biomedicina/IDISCAM, Universidad de Castilla-La Mancha/CSIC/SESCAM, C/Dr. José María Sánchez Ibáñez, S/N, 02071 Albacete, Spain; marialuisa.nueda@uclm.es; 3 Área de Bioquímica y Biología Molecular, Departamento de Química Inorgánica, Orgánica y Bioquímica, Facultad de Medicina de Albacete/IB-UCLM/Unidad de Biomedicina/IDISCAM, Universidad de Castilla-La Mancha/CSIC/SESCAM, C/Almansa 14, 02008 Albacete, Spain

**Keywords:** NOTCH, GSIs, PDAC, GC, NSCLC, TNBC, metastatic melanoma

## Abstract

**Highlights:**

**What are the main findings?**

- γ-secretase inhibitors (GSIs) and other alternative strategies demonstrate promising antitumor activity *in vitro* and in mouse xenograft models, potentiating the effects of chemotherapy and radiotherapy, and helping overcome therapy resistance and improve patient prognosis.
- Some GSIs may also exhibit dose- and time-dependent influences on the tumor’s oncogenic properties. However, despite encouraging preclinical findings, clinical trial results remain limited.

**What are the implications of the main findings?**

- The broad inhibition of the NOTCH pathway by GSIs can unintentionally suppress tumor-suppressive NOTCH receptors (such as NOTCH2 in certain breast cancer subtypes). Moreover, partial, or low-level pathway inhibition may paradoxically promote cellular proliferation, leading to unpredictable therapeutic outcomes. For these reasons, next-generation approaches should focus on developing receptor-specific GSIs or alternative NOTCH-targeting agents (e.g., DLK1/DLK2 modulators). The use of cell lines with artificially overactivated NOTCH signaling may not fully reflect the heterogeneity of human tumors, and GSIs may only target specific cellular subpopulations. In clinical settings, their application has been limited by significant toxicity and poor tolerability.
- Future research should further investigate microenvironment-driven mechanisms of drug resistance, including EMT, invasion, and stromal interactions. A deeper understanding of immune-evasion strategies could improve immunotherapy efficacy. In parallel, anti-angiogenic approaches should be prioritized as key therapeutic strategies. Advanced therapeutic modalities, such as CRISPR-based editing, CAR T-cell therapy, bispecific antibodies, and nanoparticle-mediated targeted delivery, may enhance treatment precision while reducing toxicity. Targeting cancer stem cells remains a central objective. Treatment optimization should incorporate patient stratification based on NOTCH receptor and ligand expression, pathway activation status, and immune antigen profiles. Emerging tools such as AI and big-data analytics will support the personalization of cancer therapies and should account for sex-specific biological differences to maximize therapeutic efficacy.

**Abstract:**

**Background.** NOTCH receptors play a pivotal role in carcinogenesis. Upon ligand binding, a cascade of proteolytic cleavages mediated by ADAM proteases and the γ-secretase complex activates the receptor, ultimately releasing the NOTCH intracellular domain (NICD). NICD translocates to the nucleus, where it regulates gene expression. This review mainly aims to evaluate γ-secretase inhibitors (GSIs) as anticancer agents in preclinical and clinical settings, with a focus on their ability to block tumor progression, target cancer stem cells, and overcome resistance to standard therapies. **Methods.** A systematic search was conducted in the ISI Web of Science, PubMed, and Scopus databases, following PRISMA guidelines. The review included preclinical *in vitro* and *in vivo* studies, as well as clinical trials, investigating GSIs, either as monotherapy or in combination with other treatments, in TNBC, metastatic melanoma, PDAC, gastric cancer, and NSCLC. Exclusion criteria included duplicates, non-English articles, studies published before 2010, studies on non-cancer conditions, research unrelated to NOTCH signaling, and studies outside the selected cancer types. Overall, 69 articles were included and categorized into the five types of cancer analyzed (20 on NSCLC, 22 on TNBC, 11 on metastatic melanoma, 7 on GC, and 9 on PDAC). Of these, 60 studies corresponded to preclinical research in the types of cancer, and 9 studies corresponded to clinical trials in the types of cancer except for GC. Two independent authors screened and extracted relevant data, with disagreements resolved by the corresponding author. Findings were synthesized qualitatively across cancer types under study. **Results.** This review summarizes therapeutic advances involving GSIs in cancers driven by oncogenic NOTCH signaling, based on the 69 articles included. Preclinical studies show that GSIs synergize with chemotherapy and radiotherapy, particularly in NSCLC, melanoma, and TNBC, and block EMT, overcome therapeutic resistance, and improve prognosis. Commonly used GSIs include DAPT and RO4929097, which enhance the efficacy of agents, such as gemcitabine (PDAC), paclitaxel, osimertinib, erlotinib, and crizotinib (NSCLC), and 5-FU (gastric cancer, TNBC). Promising strategies include combining GSIs with SAHA, ATRA, CB-103, and other NOTCH signaling targeting molecules, either alone or with chemo- and radiotherapy. Clinical trials with GSIs, however, remain limited. RO4929097 is the most extensively tested GSI in clinical settings. PDAC trials combining GSIs with gemcitabine showed no benefit; melanoma trials yielded modest outcomes; and TNBC trials demonstrated partial responses to GSIs but overall low efficacy and significant adverse events. **Discussion and Conclusions.** Despite encouraging preclinical evidence, clinical trials with GSIs have underperformed, largely due to tumor heterogeneity, dosing limitations, and the non-selective nature of γ-secretase inhibition. Other NOTCH inhibitors, such as DLL4 antibodies, also resulted in partial responses and secondary effects. Future strategies should prioritize receptor-specific NOTCH inhibitors, patient stratification based on NOTCH pathway activation, and optimized combination regimens. Emerging approaches include integrating immunotherapy with advanced technologies such as CRISPR, CAR-T cells, and bispecific antibodies, as well as targeted delivery systems to enhance efficacy and reduce toxicity. Additional research directions include addressing the tumor microenvironment and EMT-driven resistance, elucidating the mechanisms of immune evasion, and inhibiting tumor angiogenesis. Finally, leveraging artificial intelligence and big-data-driven personalized medicine, including sex-specific considerations, will be essential for improving patient outcomes.

## 1. Introduction

### 1.1. Global Cancer Epidemiology Overview

Cancer remains one of the leading causes of death worldwide. According to estimates from the International Agency for Research on Cancer [1], based on the most reliable data available across countries in 2022, the global burden of cancer continues to grow. That year, there were approximately 20 million new cancer cases and 9.7 million cancer-related deaths [2]. Looking ahead, the projected number of new cancer cases worldwide between 2022 and 2040, across males and females, is expected to reach 29.9 million. During the same period, the estimated number of cancer-related deaths is anticipated to rise to 15.3 million.

**Figure 1:**
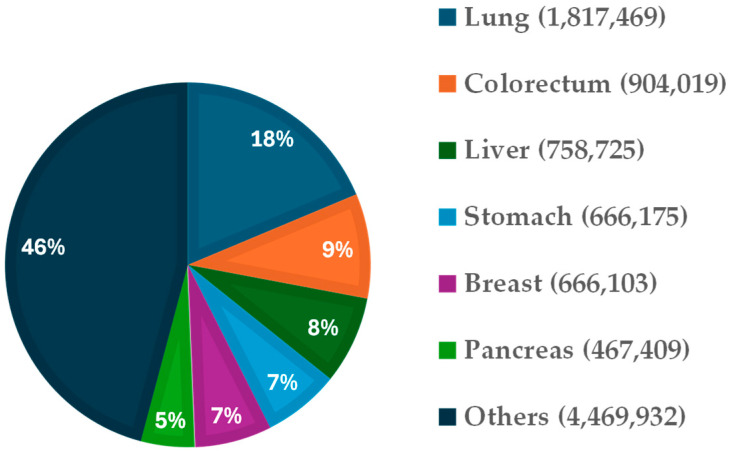
**Absolute numbers and percentages of cancer-related deaths worldwide in 2022, for males and females and all age groups.** Data analyzed by authors from [1].

According to the World Health Organization, and excluding non-melanoma skin cancers, the most frequently diagnosed tumors worldwide are breast cancer, followed by lung, colorectal, prostate, and stomach cancers [Available in [2]]. In terms of mortality, the most lethal cancers, ranked in descending order, are lung, colorectal, liver, stomach, breast, and pancreas cancer [Available in [1,2]] (Figure 1).

The molecular and cellular mechanisms driving cancer development across different tissues and organs are highly complex. Intensive global research efforts, encompassing both basic and clinical studies, are focused on elucidating these mechanisms. Such investigations are essential for the development of novel therapeutic strategies that are not only more effective but also tailored to individual patients, with reduced adverse effects.

Among the many intracellular signaling pathways implicated in cancer, the NOTCH receptor signaling pathway stands out due to its pivotal role in the initiation and progression of various neoplasms [3,4,5]. Aberrant NOTCH signaling contributes to several hallmark features of cancer and is particularly associated with the emergence of highly aggressive tumors with poor prognosis [6,7]. Moreover, the NOTCH pathway forms an intricate network of interactions with other key signaling pathways involved in oncogenesis, further driving tumor development and progression in multiple malignancies [3,8].

### 1.2. Structure of NOTCH Receptors and Their Ligands

NOTCH receptors and their ligands constitute a highly conserved protein family across evolution [9,10,11]. From a biological standpoint, the NOTCH receptor signaling pathway acts as a key regulator in cell fate decisions, controlling cellular proliferation and differentiation, senescence, or apoptosis of progenitor and stem cells, among other biological processes [7,12]. Its function is critical in numerous cellular contexts such as neurogenesis, angiogenesis, hematopoiesis, epithelial morphogenesis, and the specification of cell lineages in organs like the digestive system, lungs, skin, and immune system, and in cancer [3,7]. The NOTCH signaling pathway also plays a central role in maintaining the identity and plasticity of adult stem cells and in establishing spatial and temporal patterns of cell differentiation [7,12]. Furthermore, its involvement in phenomena such as epithelial–mesenchymal transition (EMT) and its interaction with other intracellular signaling pathways reinforces its importance in many complex biological processes in both healthy and pathological states, such as cancer [3,8]. In mammals, the main members of this protein family, both receptors and ligands, are differentially expressed depending on the cell type, physiological context, or tumor microenvironment, participating in essential cell-to-cell interactions that modulate cell fate [13,14,15].

**Figure 2:**
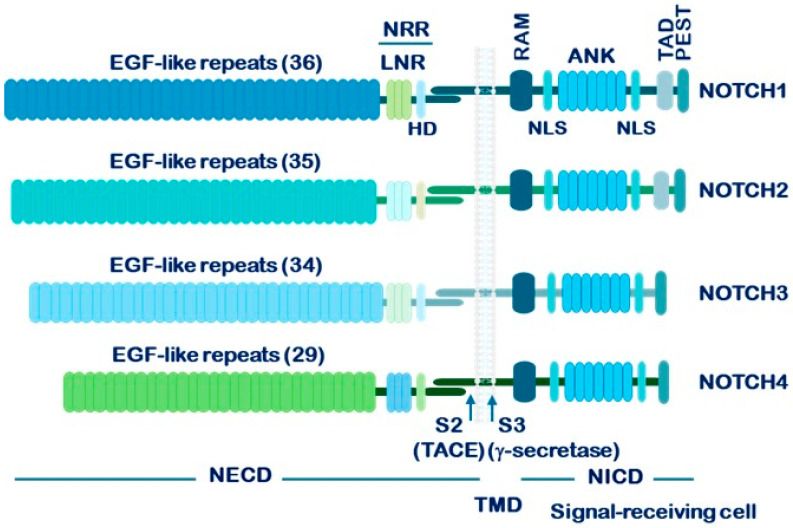
**Structure of NOTCH receptors in mammals.** The four NOTCH receptors differ primarily in the number of EGF-like repeats, with NOTCH1 and NOTCH2 containing the highest number (36 and 35 repeats, respectively). The EGF-like repeats 11–12 of NOTCH receptors interact with the DSL (Delta/Serrate/LAG2) domain of canonical activating ligands. The Negative Regulatory Region (NRR) consists of three LNR repeats and a heterodimerization (HD) domain. The NOTCH intracellular domain (NICD) includes the RAM domain, ankyrin repeats (ANK), nuclear localization signals (NLS), and the PEST domain. The S2 (Ala1710-Val1711) and S3 (Gly1753-Val1754) sites represent the proteolytic cleavage points involved in receptor processing and activation at the plasma membrane upon ligand interaction.

In mammals, unlike in Drosophila melanogaster, where the first Notch gene was discovered [16,17,18], there are four NOTCH receptors: NOTCH1, NOTCH2, NOTCH3, and NOTCH4 (Figure 2) [7,16,19,20,21].

NOTCH1 and NOTCH2 are broadly expressed across adult tissues, while NOTCH3 and NOTCH4 expression is found in vascular smooth muscle, endothelial cells, and different types of immune cells [7,13,14,15,16,21,22,23]. NOTCH receptors are heterodimeric transmembrane proteins composed of three main regions: an extracellular domain (NECD), a transmembrane domain (TMD), and an intracellular domain (NICD) [8,24,25,26]. The NECD contains multiple EGF-like repeats (epidermal growth factor-like), the number of which varies depending on the receptor subtype, and some of them specifically interact with the canonical ligands. Adjacent to the transmembrane domain, the NECD also includes a segment known as the negative regulatory region (NRR), composed of Lin12/NOTCH repeats. The active NICD region includes several functional domains: a RAM domain for protein binding, seven ankyrin repeat domains, nuclear localization signals, a transcriptional activation domain (TAD), and a PEST domain involved in protein degradation. There are five canonical activating ligands of NOTCH receptors: Delta-Like 1 (DLL1), Delta-Like 3 (DLL3), and Delta-Like 4 (DLL4), three homologs of the Drosophila Delta ligand, and Jagged1 (JAG1) and Jagged2 (JAG2), which are homologs of the Drosophila Serrate ligand [21,27,28,29,30,31] (Supplementary Figure S1). These ligands are transmembrane proteins, and their interaction with NOTCH receptors requires direct intercellular contact. Specifically, the binding occurs between specific EGF-like repeats on the NOTCH receptors and the DSL (Delta/Serrate/LAG2) domain present in the canonical ligands. In addition to canonical activating ligands, several non-canonical ligands with inhibitory functions have been identified, including DLK1 and DLK2 proteins (Supplementary Figure S2) [32,33,34,35]. DLK1 and DLK2 proteins possess six EGF-like repeat sequences in their extracellular region, a transmembrane region, and a short intracellular region [32,34]. The extracellular region of DLK1 is recognized by the TACE protease, which processes DLK1 and releases a soluble form containing the extracellular region [36,37,38,39]. DLK2 might be processed similarly to DLK1, although there is no evidence to confirm or refute this possibility. Despite lacking the DSL domain that canonical ligands possess at the N-terminal end, DLK1 and DLK2 can interact with NOTCH receptors through their N-terminal DOS domains and function as non-canonical inhibitory ligands, competing with canonical ligands [33,35,40,41]. Regarding their tissue distribution, DLK proteins also show differences. DLK1 expression in adults is limited to the adrenal glands, while DLK2 has a broader expression in adults, being mainly expressed in the skin, prostate, esophagus, brain, and salivary glands, although its expression is not remarkably high [42,43].

### 1.3. Mechanism of NOTCH Receptor Activation and Downstream Signaling

To understand the functional dynamics of NOTCH receptors, it is essential to recognize that these receptors autonomously initiate a signaling cascade that culminates in the regulation of gene expression within the nucleus [6,12,44]. Activation of NOTCH receptors involves three sequential proteolytic cleavages at conserved sites known as S1, S2, and S3–S4 [8,11,15,45,46]. The first cleavage occurs during receptor maturation in the Golgi apparatus, where the enzyme furin processes the receptor at the S1 site. This event generates a heterodimer composed of an extracellular domain, a transmembrane, and an intracellular domain, held together by non-covalent interactions. Once the receptor is transported to the plasma membrane, it remains inactive due to the presence of the Negative Regulatory Region (NRR) [47,48].

**Figure 3:**
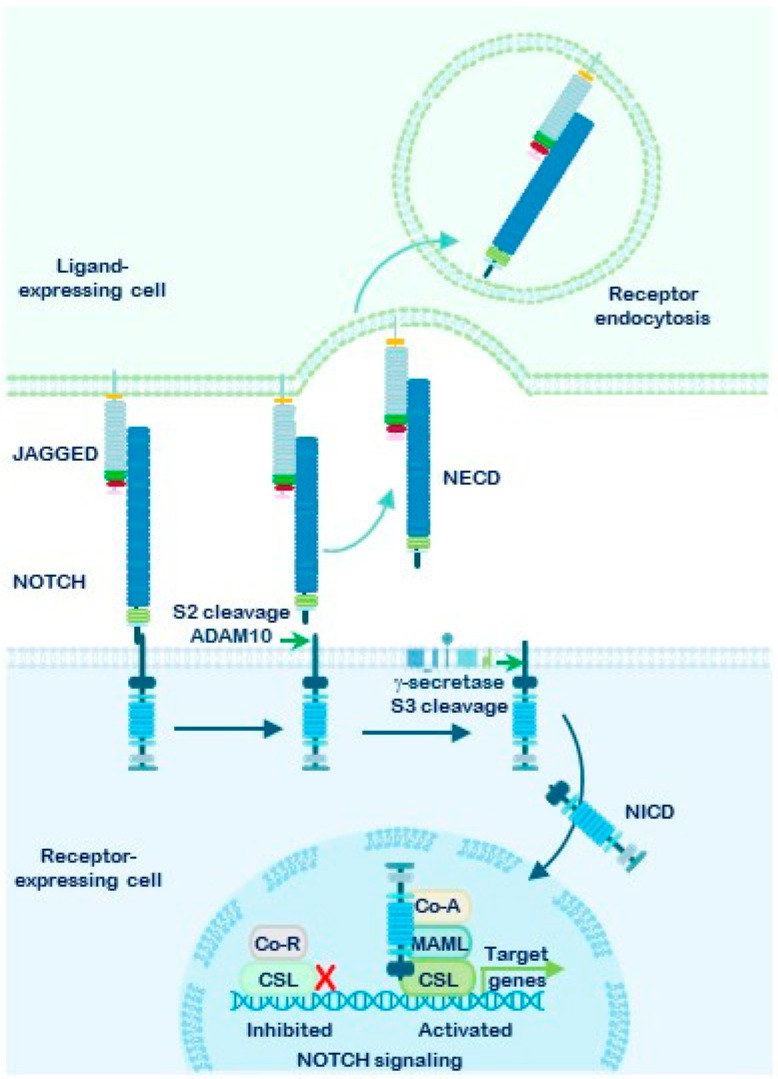
**Canonical NOTCH receptor signaling pathway.** This figure depicts the sequential proteolytic processing of a NOTCH receptor at cleavage sites S2 and S3–S4, culminating in the release of the active form, NICD (NOTCH intracellular domain). Once released, NICD translocates to the nucleus, where it interacts with transcriptional regulators to activate gene expression. Abbreviations: Co-R: co-repressor; Co-A: co-activator; CSL: CSL/RBPJκ transcription factor (CBF1/Suppressor of hairless/Lag1/Recombination signal binding protein-Jκ); MAML: Mastermind-like proteins. Red cross means inhibition of gene expression in absence of NICD.

Activation is triggered by the interaction of a canonical ligand, expressed on the membrane of a neighboring cell, with the NOTCH receptor. This ligand–receptor binding exposes the S2 cleavage site, allowing ADAM10 or ADAM17 secretases to cleave the receptor. As a result, most of the extracellular domain is endocytosed by the ligand-expressing cell [47,48] (Figure 3). This S2 cleavage is the only ligand-dependent step and represents a critical event in the canonical NOTCH signaling pathway. Following S2 cleavage, the remaining membrane-bound fragment, known as NEXT (NOTCH extracellular truncation), undergoes further processing at the S3–S4 sites by the γ-secretase complex. This final cleavage releases the active NOTCH intracellular domain (NICD), which translocates to the nucleus [15,49,50,51,52].

Within the nucleus, NICD binds to the transcription factor CSL/RBPJκ (C-promoter-binding factor, CBF1 (in mammals; also known as Recombination Signal Binding Protein Jκ, RBPJκ)/Suppressor of hairless (in *Drosophila melanogaster*)/Lag1 (in *Caenorhabditis elegans*)) along with Mastermind-like (MAML) proteins and other cofactors. This complex displaces transcriptional repressors and forms the NOTCH Transcriptional Complex (NTC), which activates the expression of target genes, including members of the *Hes* and *Hey* families (Hairy and Enhancer-of-Split) [8,44,45,53,54]. These transcriptional responses define the canonical NOTCH signaling pathway.

In addition to the canonical pathway, NOTCH signaling can also proceed via a non-canonical route, in which the NICD interacts with other intracellular proteins such as NF-κB, the serine-protein kinase ATM, or RAC1 (RAS-related C3 botulinum toxin substrate 1), among many others [3,8,55].

### 1.4. Role of NOTCH Receptors and NOTCH Ligands in Carcinogenesis

Abnormalities in the NOTCH receptor signaling pathway are well-established contributors to carcinogenesis [3,4,5,6,7]. Depending on the context, aberrant NOTCH activation can promote tumor initiation and progression, functioning as an oncogene, or conversely, its inactivation can lead to tumor development, acting as a tumor suppressor. The dual role of NOTCH signaling is highly dependent on the specific receptor involved and the tumor type. For instance, NOTCH1 may act as an oncogene in certain hematological malignancies while serving a tumor-suppressive role in skin cancers [3,4,6,55]. NOTCH3 also has a critical role in the developmental and functional pathways of immune cells and in sustaining oncogenic programs [56,57]. Despite extensive research, the molecular mechanisms underlying these opposing effects remain poorly understood. Elucidating these mechanisms is critical for the development of targeted anticancer therapies. Such therapies would aim to selectively inhibit the NOTCH receptor exhibiting oncogenic activity in a specific tumor type or counteract its role in mediating resistance to conventional chemotherapeutic agents.

NOTCH receptors can contribute to neoplastic transformation through three distinct mutational patterns [3,6,58]. The first mechanism involves chromosomal translocations that eliminate the Negative Regulatory Region (NRR), leading to constitutive activation of the receptor. This mechanism has been identified in triple-negative breast cancer. The second pattern consists of mutations in the PEST domain, which impair NICD degradation and prolong its activity. The third pattern includes mutations in the N-terminal region of the receptor, commonly observed in squamous cell carcinomas such as esophageal and lung cancers.

**Figure 4:**
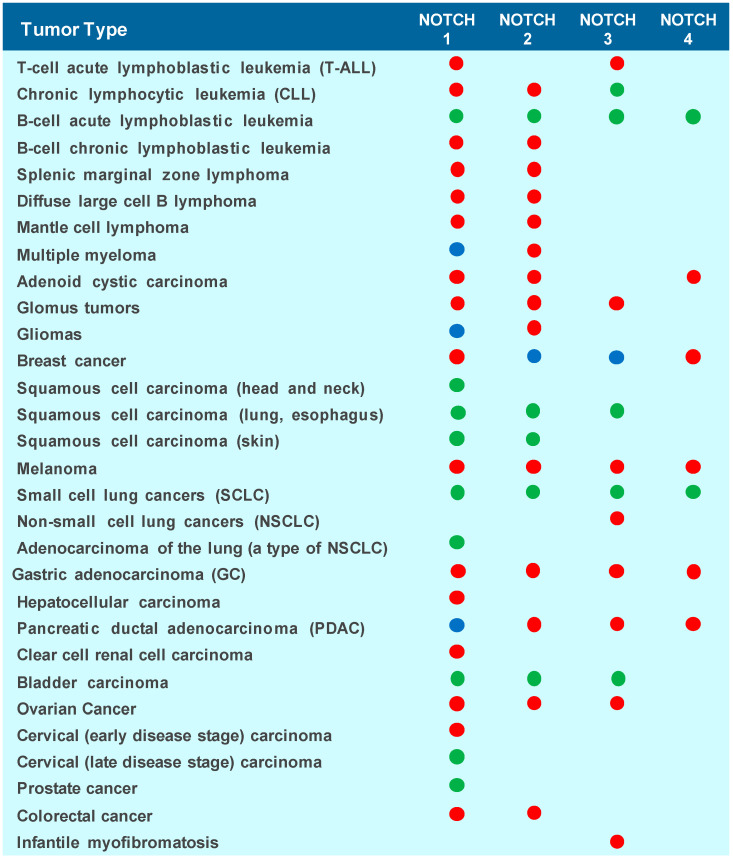
**NOTCH receptors in human cancer.** Various cancer types are classified based on whether NOTCH functions predominantly as an oncogene or as a tumor suppressor. Red circle: oncogenic role; green circle: tumor suppressor role; blue circle: oncogenic and tumor suppressor roles. Analysis made by authors from [5,7,54,59,61,62,63,64].

Beyond point mutations, two additional mechanisms can contribute to NOTCH receptor dysregulation: gene amplification and chromosomal rearrangements [6]. Gain-of-function alterations in NOTCH signaling have been associated with several cancers, including breast cancer and non-small-cell lung cancer (NSCLC). Conversely, loss-of-function mutations, where NOTCH acts as a tumor suppressor, are primarily linked to squamous cell carcinomas of the esophagus and lung [55] (Figure 4). In triple-negative breast cancer, activation of NOTCH1 has been associated with increased tumor aggressiveness, in contrast to NOTCH2-4, which may play more protective roles [59]. In highly aggressive cancers, the *Int3* oncogene, a truncated form of the NOTCH4 receptor, has also been implicated in tumorigenesis [3,60].

Beyond its role as an oncogene or tumor suppressor, NOTCH signaling plays a critical role in different processes that lead to the generation and development of tumors (Supplementary Figure S3). NOTCH signaling plays a role in the tumor microenvironment, particularly in the maintenance of cancer stem cells [6,65]. For example, in gastric cancer, overactivation of NOTCH signaling has been observed in tumor stem cell populations [66]. Increasing evidence has shown that the activation of the γ-secretase/NOTCH pathway is a key driver of drug resistance development. In breast cancer, NOTCH signaling induces cell cycle arrest in stem cells, promoting the emergence of chemoresistance [6]. NOTCH activity has also been linked to protection against drugs targeting the estrogen receptor or the MAPK pathway in certain melanomas and breast cancers [3].

NOTCH signaling is also involved in tumor infiltration and metastasis, particularly through its role in the epithelial–mesenchymal transition (EMT). During EMT, NOTCH activation leads to the downregulation of cell adhesion proteins such as E-cadherin, facilitating tumor cell migration and invasion [6]. Additionally, NOTCH receptors contribute to immune evasion, metabolic reprogramming, apoptosis inhibition, and tumor-associated inflammation [67]. In the context of angiogenesis, NOTCH canonical ligands exhibit opposing roles.

The canonical ligand DLL4 suppresses angiogenic sprouting, whereas the canonical ligand JAG1 promotes angiogenesis, tumor growth, and the maintenance of cancer stem cells. In pancreatic ductal adenocarcinoma (PDAC), NOTCH overactivation has been linked to enhanced angiogenic sprouting [68]. Elevated JAG1 expression has also been reported in breast, lung, and pancreatic cancers [69]. DLK1 and DLK2 are non-canonical NOTCH ligands that inhibit NOTCH signaling and are emerging as promising targets for developing more selective cancer therapies. DLK proteins show both oncogenic and tumor-suppressive activities, depending on the tumor type [40,70,71,72,73,74,75,76]. Although rarely expressed in normal adult tissues, DLK1 is highly overexpressed in multiple cancers, including endocrine and neuroendocrine tumors, hepatoblastoma, hepatocellular carcinoma, small-cell and non-small-cell lung cancer, pancreatic, ovarian, and brain tumors, melanoma, and TNBC [76,77,78,79,80,81], where it is often linked to poor prognosis and, in some cases, acts as a cancer stem cell marker [40]. However, anti-oncogenic roles for DLK1 have also been reported in several cancers [70,82,83,84]. DLK2 is expressed in normal adult tissues and has a role in metastatic melanoma and breast cancer [74,75,85].

### 1.5. Strategies for the Inhibition of NOTCH Receptor Signaling

**Figure 5:**
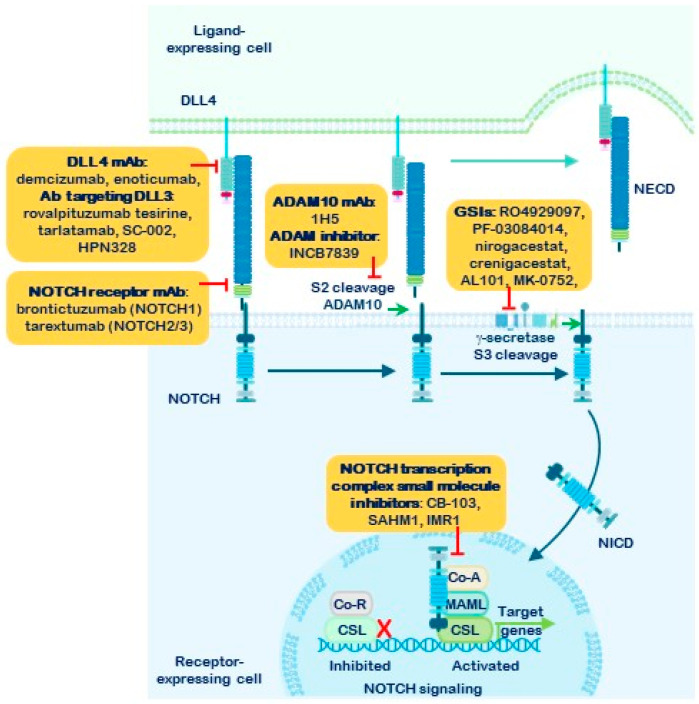
**Strategies for Inhibiting NOTCH Receptor Signaling.** This figure illustrates several therapeutic strategies designed to modulate NOTCH signaling. These include immunological approaches using antibodies that target NOTCH receptors and their ligands, chemical inhibition of ADAM proteases and the γ-secretase complex, and disruption of transcriptional regulators such as CSL (CBF1/Suppressor of Hairless/LAG-1, also known as RBP-Jκ) and Mastermind-like proteins. Collectively, these interventions aim to suppress the transcription of NOTCH target genes. All red arrows and X symbol mean inhibition.

Extensive evidence supports the hypothesis that NOTCH signaling is one of the most promising therapeutic targets in cancer treatment. In recent years, various pharmacological strategies have been developed to inhibit this pathway [86,87,88,89]. Key approaches to disrupting NOTCH receptor signaling include monoclonal antibodies (mAb) that block the receptors or their canonical and non-canonical ligands; peptides that interfere with the transcriptional activation complex; inhibitors of the α-secretases ADAM10 and ADAM17; and γ-secretase inhibitors (GSIs), which prevent the final proteolytic processing of NOTCH receptors. GSIs specifically target the γ-secretase complex, thereby blocking the release of the active NOTCH intracellular domain (NICD) (Figure 5).

### 1.6. The γ-Secretase Complex and γ-Secretase Complex Inhibitors (GSIs)

The γ-secretase complex (GSC) is a member of the intramembrane-cleaving proteases (I-CLiPs) family [50]. It plays a central role in the proteolytic processing of several substrates, including NOTCH receptors and the β-amyloid precursor protein, whose cleavage generates the β-amyloid peptide implicated in the formation of senile plaques in Alzheimer’s disease [86], and E-cadherin, a key molecule in cell adhesion [90]. Beyond its proteolytic functions, the GSC also participates in non-proteolytic processes such as calcium homeostasis, autophagy, and apoptosis [91], earning it the nickname “Transmembrane Cellular Proteasome”.

**Figure 6:**
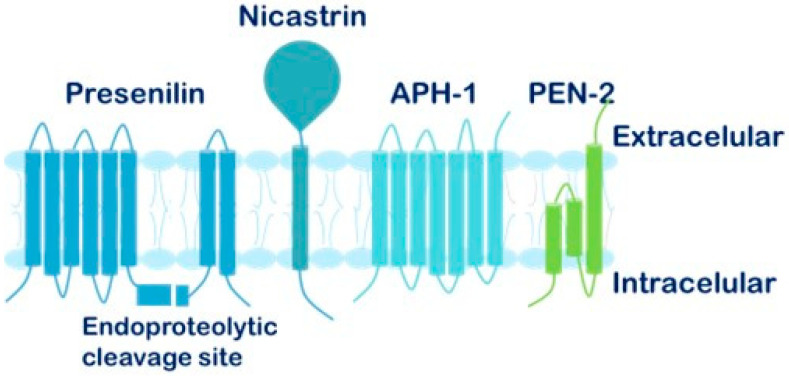
**Schematic representation of the γ-secretase complex (GSC).** This diagram illustrates the structural organization of the γ-secretase complex, with each component traversing the membrane multiple times. Presenilin, the catalytic core of the complex, is depicted as comprising two subunits, and it is essential for the proteolytic activity of the enzyme.

The GSC is composed of several transmembrane subunits (Figure 6): Presenilin (PS), with two isoforms (PS1 and PS2), is responsible for the catalytic activity of the complex [91,92]; Nicastrin (NCT) acts as a scaffolding protein that recognizes and binds substrates targeted for cleavage; Anterior pharynx defective 1 (APH-1), which exists in multiple isoforms, contributes to complex stabilization [93]; and Presenilin enhancer 2 (PEN-2) is involved in the heterodimerization of presenilin and the maturation of nicastrin.

γ-secretase inhibitors (GSIs) were initially developed as therapeutic agents for Alzheimer’s disease [86,94,95]. However, their clinical use was discontinued due to limited efficacy and significant adverse effects, including the emergence of non-melanoma skin tumors. Interest in GSIs was later rekindled in the field of oncology, driven by the role of NOTCH receptors in tumorigenesis. Currently, extensive research is underway to evaluate GSIs as potential anti-cancer agents [96,97,98]. Both preclinical and clinical studies have explored their ability to suppress tumor progression by targeting NOTCH signaling pathways *in vitro* and *in vivo*. Moreover, GSIs may hold promise in overcoming chemotherapy resistance by modulating the γ-secretase/NOTCH axis. In addition, GSIs are being investigated as candidates for therapies targeting cancer stem cells (CSCs), which are characterized by slow proliferation and resistance to conventional chemotherapy and radiotherapy, factors that contribute to treatment failure and disease recurrence. Eradicating CSCs is considered a key strategy for achieving long-term cancer remission. Promising studies have shown that GSIs, when combined with other anticancer agents, exert a stronger inhibitory effect on CSCs [99].

It is important to note that GSIs exhibit differential inhibition profiles across various NOTCH substrates. In some cases, GSIs enhance the cleavage of certain NOTCH substrates at concentrations that inhibit NOTCH1 cleavage [100]. This paradoxical effect may result from the direct action of low GSI concentrations on γ-secretase itself.

Nonetheless, numerous side effects have been reported in clinical trials, largely due to GSIs’ broad activity across multiple substrates and biological processes. For this reason, gamma-secretase inhibitors (GSIs) are associated with several side effects that remain a subject of debate among researchers [101]. The most common adverse effects include gastrointestinal toxicity (diarrhea, nausea, and vomiting) due to disruption of NOTCH signaling and skin toxicity linked to impaired protein production for skin health. These symptoms are generally mild to moderate and manageable. Concerns about neurological effects exist, but clinical trials have not reported significant findings to date. Other reported side effects include fatigue, headache, and anemia. Overall, while GSIs show therapeutic potential, their risks must be carefully balanced against benefits, and further research is required to establish their safety and efficacy [96].

This review explores therapeutic strategies involving gamma-secretase inhibitors (GSIs) and some other alternatives, administered either as monotherapy or in combination with other agents, in five highly aggressive malignancies: non-small-cell lung cancer (NSCLC), triple-negative breast cancer (TNBC), metastatic melanoma, gastric cancer, and pancreatic ductal adenocarcinoma (PDAC), all characterized by oncogenic NOTCH signaling.

## 2. Methods

**Figure 7:**
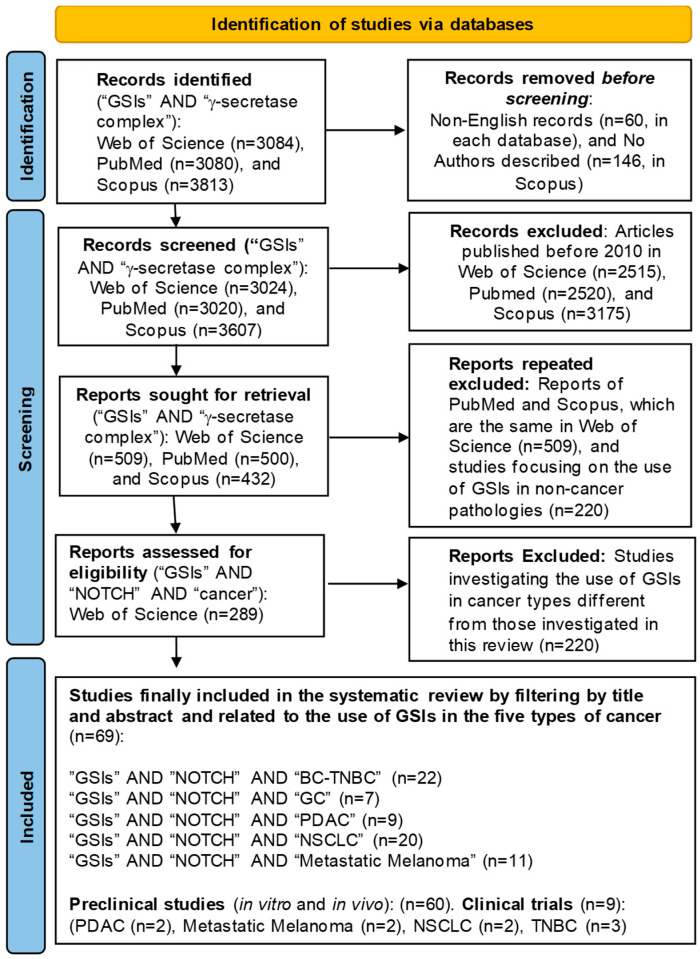
**Flow diagram of the systematic review.** This diagram illustrates the database search process and the number of publications identified and screened, and the final full-text articles included in the systematic review. The review evaluated the main therapeutic advances achieved with GSIs *in vitro*, *in vivo*, and in clinical trials across five highly aggressive cancers driven by oncogenic NOTCH signaling: Pancreatic Adenocarcinoma (PDAC), Triple-Negative Breast Cancer (TNBC), Non-Small-Cell Lung Cancer (NSCLC), metastatic melanoma, and Gastric Cancer (GC).

This systematic review was performed to investigate the recent findings that underscore advances in GSI-based therapies and some other alternatives against five aggressive malignancies (triple-negative breast cancer (TNBC), metastatic melanoma, pancreatic ductal adenocarcinoma (PDAC), gastric cancer (GC), and non-small-cell lung cancer (NSCLC)), based on the preferred reporting items for systematic review and meta-analysis (PRISMA) (Supplementary Materials) [102]. Details are presented in Figure 7. The study was not registered because it is a mixed qualitative revision that includes *in vitro* research, animal model studies, and a small number of human clinical trials.

### 2.1. Information Source and Search Strategy

All English publications investigating GSI-based and some other alternative therapies for the five aggressive malignancies under study were searched using the online databases ISI Web of Science (http://www.webofknowledge.com) (access dates: 15 January 2025 and 30 January 2026), PubMed (https://pubmed.ncbi.nlm.nih.gov/) (access dates: 20 January 2025 and 30 January 2026), and Scopus (https://www.scopus.com/) (access dates: 30 January 2025 and 30 January 2026) to identify relevant studies published between 1 January 2010 and 31 January 2026. These studies were revised by three independent authors: Pablo Martínez-Gascueña (P.M.-G.), María-Luisa Nueda (M.-L.N.), and Victoriano Baladrón (V.B.). The following search terms were used for systematic search in the title and abstract: “GSI,” “gamma-secretase complex,” “cancer,” “NOTCH,” “pancreatic adenocarcinoma (PDAC),” “triple-negative breast cancer (TNBC),” “metastatic melanoma,” “non-small cell lung cancer (NSCLC),” and “gastric cancer (GC)”. The initial search employed the keywords “GSIs (gamma-secretase inhibitors)” AND “gamma-secretase complex” across the three databases. After applying preliminary exclusion parameters, the keywords “GSIs” AND “NOTCH” AND “cancer” were used. Once eligible studies from the Web of Science database addressing GSIs in cancer and their relationship to the NOTCH signaling pathway were identified, articles were further filtered using the keywords “GSIs” AND “NOTCH” AND each of the five cancer types under study. Finally, a total of 69 articles were included and categorized into the five types of cancer analyzed (20 on NSCLC, 22 on TNBC, 11 on metastatic melanoma, 7 on GC, and 9 on PDAC). Of these, 60 studies correspond to preclinical research in the types of cancer, and 9 studies correspond to clinical trials in the types of cancer except for GC (Figure 7). Additional references, including those before 2010, were consulted for background information, including reviews on NOTCH signaling, GSIs in cancer, and emerging therapeutic approaches, which informed the Introduction and Discussion sections.

### 2.2. Eligibility Criteria

Original articles with these criteria were included: preclinical research conducted *in vitro* and *in vivo* using animal models, as well as clinical trials in humans evaluating GSIs, and some other alternatives, either as monotherapy or in combination with other anticancer agents, targeting TNBC, metastatic melanoma, PDAC, GC, and NSCLC.

Exclusion criteria were articles published before 2010, non-English publications, studies with unknown authors in Scopus, and studies investigating GSIs in non-cancer pathologies or cancer types outside the scope of this review.

### 2.3. Data Extraction

All duplications were removed, and the remaining articles were reviewed by the three authors independently. Subsequently, titles and abstracts were screened to retain studies directly related to GSIs in the selected cancer types. Then, the full text of all studies was evaluated, and selected studies were thoroughly read by two independent authors (P.M.-G. and M.-L.N.), who extracted the most relevant information into a notebook. Any disagreements were resolved by the final author (V.B.). Finally, the most relevant information was incorporated into the Results section of the manuscript and discussed in the Discussion section. Findings were synthesized qualitatively across the selected cancer types.

In this review, we did not conduct a formal risk-of-bias assessment using structured tools such as ROBINS-I, SYRCLE, RoB, or OHAT. Our primary aim was to provide a descriptive and exploratory synthesis of the available literature rather than a graded appraisal of causal inference or a quantitative meta-analysis.

## 3. Results

The next subsections in this section analyze the recent findings that underscore advances in GSI-based therapies for the five aggressive malignancies under study with poor prognosis. 69 articles were included and categorized into the five types of cancer analyzed (Figure 7). Evidence from both preclinical studies and clinical trials highlights the potential of GSIs and alternative molecules, particularly when combined with established anticancer agents, to improve therapeutic efficacy and reduce adverse effects associated with their nonspecific mechanisms of action.

### 3.1. The Combination of GSIs with Other Therapeutic Agents Has Demonstrated Efficacy in Reducing Pancreatic Ductal Adenocarcinoma (PDAC) Progression in Preclinical Studies and Clinical Trials

The development of pancreatic ductal adenocarcinoma (PDAC) proceeds through well-defined precursor stages, most notably acinar-to-ductal metaplasia (ADM), and pancreatic intraepithelial neoplasia (PanIN). A hallmark molecular event in this progression is the acquisition of activating mutations in KRAS, which are detected in the vast majority of PanIN lesions and are considered indispensable for both malignant transformation and subsequent evolution toward invasive PDAC [103]. Genetically engineered mouse models with conditional KRAS activation faithfully recapitulate PanIN initiation and progression. Notably, although KRAS activation occurs broadly in pancreatic epithelial cells in these models, only a restricted subset acquires the competence to form PanINs [104,105].

Emerging evidence indicates that NOTCH signaling exhibits context-dependent and stage-specific roles during PDAC pathogenesis. NOTCH receptors are frequently upregulated in PDAC, mediated through both canonical and non-canonical pathways [106]. Among these, NOTCH4 has been identified as a key promoter of tumorigenesis; *Notch4* ablation markedly reduces ADM and PanIN formation, delays progression to advanced PDAC, and significantly extends survival in aggressive mouse models [107]. In contrast, NOTCH1 can exert tumor-suppressive effects in early disease, as genetic deletion of *Notch1* unexpectedly accelerates PDAC development [108].

Functional studies provide further insight into this duality. NOTCH pathway activation synergizes with oncogenic KRAS to markedly enhance PanIN initiation and dramatically increase lesion burden [104,105,106,109]. Downstream mediators highlight additional layers of complexity: HES1, although dispensable for pancreatic organogenesis, is essential for maintaining adult acinar differentiation. In KRASG12D models, loss of *Hes1* augments ADM yet reduces high-grade PanIN formation while paradoxically accelerating PDAC progression [110]. Downregulation of RBPJκ, the principal transcriptional effector of canonical NOTCH signaling, derepresses NOTCH target genes and enhances KRAS-driven acinar transformation and desmoplastic remodeling [111]. Basal NOTCH activity cooperates with IKK2/NF-κB signaling to promote pancreatic carcinogenesis by augmenting NOTCH target gene expression and suppressing anti-inflammatory responses [112].

Collectively, these findings suggest that NOTCH signaling constrains early lesion formation but facilitates late-stage PDAC progression, underscoring the need for therapeutic strategies that consider disease stage. Agents such as KRAS pathway inhibitors and γ-secretase inhibitors (GSIs) may therefore confer therapeutic benefit but could exert deleterious effects during early tumorigenesis.

Conversely, the glycosyltransferase Lunatic Fringe (LFNG) acts as a potent tumor suppressor in KRAS-initiated pancreatic cancer. LFNG regulates NOTCH receptor glycosylation and modulates transforming growth factor beta (TGF-β) signaling by repressing transcription of TGF-β pathway genes during PDAC development [113]. Finally, therapeutic strategies targeting pathways downstream of mutant KRAS, including MAPK and PI3K inhibition, siRNA-mediated KRAS silencing, and enzymatic hyaluronan depletion using PEGylated hyaluronidase, have demonstrated encouraging activity in early-stage evaluations and represent promising avenues for future therapeutic development [106].

JAGGED2 canonical ligand is aberrantly overexpressed in human PDAC and uniquely drives metastatic progression, in contrast to JAGGED1. Notably, inhibition of downstream NOTCH signaling does not substantially impair metastasis, despite evidence of JAGGED2-NOTCH1 interaction [114].

DLL4 and NOTCH1 are commonly upregulated in solid tumors and linked to chemoresistance in pancreatic cancer models and have been associated with poor prognosis in lung cancers such as PDAC, as well as with advanced tumor stage and lymph node metastasis. However, evidence suggests that adjuvant therapies targeting DLL4-NOTCH signaling may improve clinical outcomes [115,116]. DLL4 expression also may help predict which patients with resected pancreatic cancer will benefit from gemcitabine treatment [117].

In relation to the use of GSIs in PDAC, Cook and colleagues evaluated the γ-secretase inhibitor MRK003 in pancreatic ductal adenocarcinoma (PDAC) xenograft models, both as a monotherapy and in combination with gemcitabine. Their results indicated that MRK003 as monotherapy did not elicit significant therapeutic effects; however, its combination with gemcitabine led to a substantial improvement in mouse survival [68]. Treatment with MRK003 was associated with increased necrosis of neoplastic tissue, enhanced apoptosis, and reduced cellular proliferation. One of the principal mechanisms attributed to MRK003 was its capacity to inhibit intratumoral vascular proliferation, observed both as monotherapy treatment and in combination therapy. This vascular suppression induced hypoxic conditions within the tumor microenvironment, thereby potentiating the efficacy of the therapeutic agents. In a separate study, Mizuma and colleagues similarly demonstrated that the combination of MRK003 and gemcitabine effectively impeded tumor progression in PDAC mouse models [118].

GSI IX treatment induced apoptosis and selectively targeted epithelial–mesenchymal transition (EMT) markers. Notably, GSI IX suppressed the growth of pancreatic tumor-initiating CD44^+^/EpCAM^+^ cells both *in vitro* and in xenograft mouse models, underscoring its potential as a therapeutic strategy against pancreatic cancer by impairing tumor initiation and limiting epithelial plasticity [98]. In other work, Palagani and colleagues explored the therapeutic potential of combining GSI-IX with AG-490, a Janus Kinase 2 (JAK2) inhibitor that blocks activation of STAT3, signaling molecules implicated in PDAC pathogenesis. Using mouse models exhibiting pancreatic intraepithelial neoplasia (PanIN) and acinar-to-ductal metaplasia (ADM), they tested each compound as monotherapy. After six weeks, small tumors and microscopic foci of PDAC were observed. Remarkably, in the group treated with the combination of GSI-IX and AG-490, none of the five mice developed visible tumors, indicating a synergistic therapeutic effect [119].

The stroma component of pancreatic cancer makes up to 90% of the tumor mass and is thought to be one of the main reasons for the tumor’s high chemoresistance. Cancer-associated fibroblasts (CAFs) have previously been identified as key stromal players. Neumann and coworkers study the effect of DAPT in PDAC cell lines and associated CAFs. Unlike PDAC cells, CAF monocultures hardly responded to any treatment, which suggested that stroma (CAFs) itself is more resistant to standard chemo-treatments than the epithelial cancer cells. Elevated levels of IL-6 were also associated with a reduced response to therapy [120].

The γ-secretase inhibitor PF-03084014 has shown promising results in a phase III clinical trial for desmoid tumors (aggressive fibromatosis), rare, non-malignant connective tissue growths that are locally invasive and prone to recurrence [121]. In the context of PDAC, Yabuuchi and colleagues assessed PF-03084014 in various xenograft mouse models, both as monotherapy and in combination with gemcitabine [109]. While PF-03084014 as monotherapy did not significantly inhibit tumor proliferation, its combination with gemcitabine resulted in notable antiproliferative effects and tumor regression. Flow cytometry analyses revealed that gemcitabine as monotherapy failed to eliminate tumor stem cells and even appeared to increase their prevalence. In contrast, PF-03084014, both as monotherapy and in combination, reduced the population of tumor stem cells. Moreover, the combination therapy significantly decreased distant metastasis, suggesting a broader impact on tumor aggressiveness and dissemination [109].

It is worth highlighting the development of the γ-secretase inhibitor (GSI) MRK-0752, which was evaluated by Cook and colleagues in a phase I clinical trial in combination with gemcitabine in patients with stage IV pancreatic ductal adenocarcinoma (PDAC). In this study, 14 out of 44 patients achieved disease stabilization. However, the outcomes were comparable to those observed with gemcitabine monotherapy [122]. Notably, several adverse effects associated with GSI treatment were reported, including gastrointestinal disturbances, thrombocytopenia, and anemia.

The γ-secretase inhibitor RO4929097 was evaluated in a phase II clinical trial involving patients with previously treated metastatic pancreatic adenocarcinoma; however, the trial was terminated due to the discontinuation of GSI synthesis [123].

Conversely, ligand-independent NOTCH inhibition has also been described. Exosomes released by SOJ-6 pancreatic tumor cells induce ligand-independent NOTCH1 inactivation and promote cell death. Synthetic exosome-like nanoparticles enriched in cholesterol disrupt the lipid architecture of membrane microdomains that host NOTCH-interacting partners, including components of the γ-secretase complex, thereby impairing NOTCH1 function [124].

Historically, gemcitabine as a single agent has been the standard treatment for pancreatic cancer. However, recent data suggest that gemcitabine plus Abraxane may be superior to gemcitabine alone, and therefore this combination is emerging as the new standard therapy for pancreatic cancer. Demcizumab is a humanized monoclonal antibody developed to target cancer stem cells. Demcizumab has been used in a phase 1b study combined with gemcitabine and/or nab-paclitaxel in patients with pancreatic cancer. This therapy was generally well tolerated but presented secondary effects. Truncated demcizumab dosing (i.e., limited to 70 days) avoided clinically significant cardiopulmonary toxicity. Another phase 1b study of gemcitabine and demcizumab (OMP-21M18) with or without Abraxane as first-line treatment in subjects with locally advanced or metastatic pancreatic cancer (NCT01189929) is also being conducted. The purpose of this study is to evaluate the safety and determine the optimal dose of demcizumab when administered in combination with gemcitabine, with or without Abraxane. These preliminary studies related to pancreatic cancer have not been included in the systematic review because they are not officially published.

### 3.2. Treatment Resistance in Non-Small-Cell Lung Cancer (NSCLC) Can Be Mitigated Through the Application of γ-Secretase Inhibitors as Monotherapy and Combined with Other Drugs

NOTCH signaling plays a critical role in the pathogenesis of non-small-cell lung cancer (NSCLC) [125]. NOTCH1 is required for tumor initiation through suppression of p53-mediated apoptosis, acting by regulating p53 stability. These findings identify NOTCH1 as a key effector in KRAS-driven lung adenocarcinoma [126]. NOTCH3 overexpression has been reported in approximately 40–50% of cases while elevated NOTCH1 levels are associated with poor prognosis and tumor initiation, particularly in KRAS-driven models [127]. Interestingly, in the squamous subtype of NSCLC, NOTCH signaling appears to be suppressed, suggesting a potential tumor-suppressive function in this context [128]. The oncogenic functions of individual NOTCH receptors *in vivo* remain only partially resolved. In a KRASG12D-driven endogenous NSCLC mouse model, Baumgart and colleagues evaluated the receptor-specific contributions of NOTCH1 and NOTCH2 through conditional gene deletion. Loss of NOTCH1 significantly reduced early tumorigenesis and attenuated MAPK signaling, whereas NOTCH2 deletion produced the opposite phenotype, resulting in a marked increase in carcinogenesis accompanied by elevated MAPK pathway activation. Consistent with these findings, ablation of NOTCH1, but not NOTCH2, reduced expression of the NOTCH targets HES1 and DUSP1, supporting a model in which NOTCH2 promotes epithelial differentiation and functions as a tumor suppressor, while NOTCH1 drives tumor initiation and malignant progression [129].

Additional studies report ligand-independent NOTCH activation in NSCLC. Approximately 30% of human NSCLC tumors exhibit loss of NUMB, an intrinsic inhibitor of NOTCH signaling [130]. Moreover, nearly 10% of patients harbor NOTCH1 gain-of-function mutations affecting the heterodimerization, transactivation, or PEST domains, leading to constitutive pathway activation [131]. The protein POGLUT-1 also modulates NOTCH activity in NSCLC; POGLUT-1 silencing substantially decreases tumor cell proliferation, migration, and survival [132].

NOTCH signaling is also essential for the development and maintenance of KRASG12V-driven NSCLC. Genetic studies demonstrate that γ-secretase and RBPJκ are required for tumor initiation, and pharmacological γ-secretase inhibition robustly suppresses tumor growth *in vivo.* HES1 represses DUSP1, a phosphatase that deactivates phospho-ERK; thus, γ-secretase inhibition via DAPT increases DUSP1 expression, reduces phospho-ERK levels, and attenuates tumor progression [133].

A specific subpopulation of tumor-propagating cells (TPCs) supports sustained tumor growth in NSCLC. The CD24^+^ITGB4^+^NOTCH^hi subset demonstrates robust tumor-initiating capacity in clonogenic and orthotopic serial-transplantation assays. Treatment with the γ-secretase inhibitor DAPT markedly decreases primary pulmospheres formation and reduces expression of NOTCH targets HES1 and HEY1, consistent with pathway blockade. Although all four NOTCH receptors are expressed in TPCs, NOTCH3 uniquely governs tumor propagation in both mouse models and KRAS-mutant human NSCLC. This TPC compartment becomes enriched following chemotherapy, and its transcriptional signature correlates with poor clinical outcomes in human tumors. These findings identify NOTCH3 as a key driver of tumor maintenance and an attractive therapeutic target in NSCLC [134].

In NSCLC, nuclear DLK1, a NOTCH inhibitory ligand, expression correlates with tumor differentiation and size. The tumor suppressor NCOR1 has been identified as a nuclear DLK1-interacting protein, suggesting that DLK1–NCOR1 cooperation may regulate cell differentiation [135].

NOTCH signaling plays a critical role in the pathogenesis and therapy resistance of non-small-cell lung cancer (NSCLC) [136], and treatment with γ-secretase inhibitors has the potential to overcome or counteract this resistance. Recent studies have demonstrated synergistic interactions between NOTCH/γ-secretase inhibition and standard-of-care therapies in NSCLC. The γ-secretase inhibitors dibenzazepine (DBZ) and BMS-906024 show strong synergy with several chemotherapeutic agents, including cisplatin, etoposide, paclitaxel, crizotinib, docetaxel, and pemetrexed, administered either alone or in combination with radiation therapy. The most potent therapeutic effect was observed with the triple-combination of crizotinib, NOTCH inhibition, and radiation [137]. In 2D cultures, BMS-906024 monotherapy produced no significant reduction in proliferation; however, its combination with paclitaxel or crizotinib, with or without radiation, resulted in a marked suppression of cell growth. In 3D spheroid models, BMS-906024 alone reduced spheroid expansion, with further enhancement when combined with crizotinib or radiation. The greatest reduction in spheroid viability occurred when all three treatments were applied simultaneously.

Collectively, these preclinical findings indicate that adding a GSI to crizotinib, either alone or together with radiation, significantly increases NSCLC spheroid sensitivity and may counteract or delay the emergence of crizotinib resistance. Clinically, crizotinib achieves partial or complete responses in approximately 61% of patients, yet most individuals develop resistance within 12 months. Future studies will be required to determine whether NOTCH inhibition can overcome established crizotinib resistance *in vivo*.

The use of BMS-906024 GSI in xenograft models also demonstrated enhanced cytotoxicity when combined with paclitaxel, particularly in tumors harboring KRAS or BRAF mutations. This effect was linked to mutant or null p53 status, contrasting with other studies that associate NOTCH1 activity with p53 expression [138,139].

Liu and colleagues demonstrated *in vitro* that cisplatin treatment of NSCLC cell lines led to the enrichment of CD133^+^ and ALDH^+^ cells, markers of lung cancer stem cells (LCSCs) linked to chemoresistance. Pre-treatment with the γ-secretase inhibitor DAPT significantly inhibited the selection of these stem-like cells and reduced cisplatin resistance [140]. Treatment with GSI-34 of a CD166^+^Lin^−^ subpopulation with LCSC characteristics and intrinsic resistance to cisplatin in xenograft mouse models sensitized these cells to cisplatin, resulting in reduced tumor size, with the most pronounced effect observed when both agents were combined [127]. Xie and colleagues investigated strategies to overcome resistance to gefitinib, an EGFR inhibitor, in NSCLC. Using gefitinib-resistant cell lines, they applied GSI BMS-708163 and found that high doses reversed resistance. In 3D cultures, treated cells formed significantly smaller colonies, with enhanced effects when combined with gefitinib. *In vivo* experiments in xenograft models confirmed that the combination significantly inhibited tumor growth [141].

Osimertinib is a third-generation EGFR-TKI used in EGFR-mutated NSCLC. Despite its strong anti-tumor activity, many patients develop resistance through drug-tolerant persisted (DTP) cells. Researchers observed that NOTCH1 and its downstream targets are upregulated in DTP cells, implicating NOTCH signaling in resistance. *In vitro* and *in vivo* experiments demonstrated that combining osimertinib with GSI XX impaired drug-tolerant persistence, suppressed phospho-ERK, and enhanced DUSP1 expression. These findings suggest that co-administration of GSIs with osimertinib may represent a promising therapeutic strategy to overcome resistance in EGFR-mutated NSCLC [142].

Mizugaki and colleagues found that NSCLC cell lines exposed to RT exhibited increased expression of NOTCH1 and NOTCH3 at 48 h. Treatment with GSI-I or GSI-XX in combination with RT led to higher levels of apoptosis compared to RT as monotherapy. In xenograft models, the combination of RT and GSI-XX significantly delayed tumor growth relative to either treatment as monotherapy [143].

GSI XX and ABT-737 independently inhibit cell proliferation in a dose-dependent manner, while their combination produces a synergistic antitumor effect *in vitro* and markedly suppresses tumor growth *in vivo*. Treatment with either agent regulated the expression of apoptosis proteins. The enhanced antitumor activity of GSI XX and ABT-737 in NOTCH-expressing non-small-cell lung cancer leads to apoptosis [144].

NSCLC frequently develops resistance to conventional chemotherapy such as paclitaxel through NOTCH signaling–mediated mechanisms. Targeting NOTCH3 with specific inhibition via GSI-IX was shown to overcome this resistance. In combination with paclitaxel, NOTCH3 inhibition produced a synergistic antitumor effect by modulating the intrinsic apoptosis pathway and enhancing cell death. Moreover, GSI-IX reduced NOTCH3–induced chemoresistance in a concentration-dependent manner [145].

Survival of NSCLC cells in hypoxic tumor environments relies on NOTCH1 signaling, which contributes to chemotherapy resistance, recurrence, and metastasis. Using an orthotopic NSCLC model, researchers inhibited the NOTCH1/IGF-1R/AKT-1 axis with three agents: the γ-secretase inhibitor MRK-003, the IGF-1R antibody MK-0646, and the pan-AKT inhibitor MK-2206. All treatments, except AKT inhibition, significantly prolonged median survival in mice. MRK-003 specifically induced cell death in hypoxic tumors, reduced hypoxia markers, and decreased metastasis to the liver and brain. Sequential administration of MK-0646 followed by erlotinib improved survival, whereas simultaneous treatment was less effective [146].

NOTCH pathway inhibition with the γ-secretase inhibitor MRK-003 reduces the clonogenic potential of cancer cell lines *in vitro*, and this effect can be reversed by expressing a constitutively active form of NOTCH3. However, *in vivo*-induced expression of a dominant-negative NOTCH pathway inhibitor shows no clear impact on tumorigenicity. These contrasting results may reflect differences between the roles of individual NOTCH isoforms versus global pathway suppression, as well as fundamental distinctions between clonogenic survival in simplified culture systems and tumorigenic capacity within complex multicellular environments [147].

The cancer stem cell model proposes that tumors contain a subpopulation of cells with self-renewal, differentiation, and tumor-initiating capacity. In lung cancer, a NOTCH-responsive GFP reporter identified a GFP-bright subset with high NOTCH activity. These GFP-bright cells formed more tumorspheres in serum-free conditions, were resistant to chemotherapy, and remained tumorigenic in serial xenotransplantation assays. Tumor xenografts from mice treated with the γ-secretase inhibitor MRK-003 showed reduced expression of downstream NOTCH effectors and failed to regenerate tumors after reimplantation into mice [148]. Multivariate analysis in a cohort of 441 lung adenocarcinoma patients revealed a significant association between high NOTCH activity, reflected by increased ligand expression or reduced expression of negative regulators, and poor clinical outcome. This relationship was independently validated in a second cohort of 89 adenocarcinoma patients, where *HES1* overexpression correlated with reduced overall survival [148].

Stromal-mediated resistance significantly limits the effectiveness of immunotherapy in lung adenocarcinoma. Single-cell RNA sequencing of over 250,000 cells from treatment-naïve patients identified tumor-enriched mesenchymal subsets *of* cancer-associated fibroblasts (CAFs) and ACTA2^+^MCAM^+^ pericytes, which interact closely with endothelial cells in the perivascular niche. Computational modeling revealed that these interactions are driven by NOTCH signaling, with CAFs and pericytes functioning as signal receivers and immature neovascular endothelial cells as signal senders. Inhibition of NOTCH signaling with GSI MRK-003 or depletion of NOTCH3 reduced collagen production and suppressed invasive behavior. A T cell–inflamed gene signature predicted survival only in patients with low NOTCH3 expression [149].

Gamma-secretase inhibitors, such as DAPT, also play a significant role in angiogenesis because the NOTCH signaling pathway regulates vascular development. In experimental models using human lung adenocarcinoma xenografts in nude mice, DAPT reduced endothelial cell proliferation without inducing cellular toxicity, suppressed capillary structure formation *in vitro*, and inhibited microvessel sprouting in the rat aortic ring assay. Collectively, these findings demonstrate that DAPT potently blocks angiogenesis, leading to impaired tumor vascularization and reduced tumor growth [150].

Challenges in applying particle therapy to NSCLC, particularly the impact of lung tissue heterogeneity on dose modulation, have also been performed. Lung modulation models into clinical workflows are critical for optimizing particle therapy outcomes in NSCLC [151].

Immune checkpoint inhibitors (ICIs) have produced substantial clinical benefit in advanced non-small-cell lung cancer (NSCLC), yet only a minority of patients achieve durable responses, and many experience ultra-rapid disease progression. Genetic and pharmacological inhibition of DDR1 suppresses tumor initiation and tumor progression, respectively. Importantly, simultaneous inhibition of DDR1 and NOTCH signaling induces regression of KRAS/TP53-mutant patient-derived lung xenografts (PDX), achieving therapeutic efficacy at least comparable to standard chemotherapy [152]. Zhang and colleagues identified co-occurring alterations in NOTCH and DNA damage response (DDR) pathways as a novel predictor of improved immunotherapy efficacy in NSCLC. Across three preliminary cohorts, patients harboring co-mutations in NOTCH and DDR genes showed superior responses to immunotherapy, and the data suggested potential benefit from combining NOTCH inhibitors with DDR inhibitors. This genomic signature offers a new dimension for predicting enhanced survival outcomes following immunotherapy [64].

Recently, evodiamine (EVO), a low-toxicity natural alkaloid, has emerged as an alternative to GSIs and as a potential antitumor agent for NSCLC. EVO reduced cell proliferation and metastasis and lowered expression of NOTCH3 and GSC. Although EVO did not bind directly to NOTCH3, it showed strong affinity for GSC, comparable to that of GSIs [153].

Another alternative is the use of antibodies to NOTCH and ligand proteins. A phase Ib trial evaluated the anti-cancer stem cell DLL4-binding agent demcizumab in combination with pemetrexed and carboplatin as first-line therapy for metastatic non-squamous NSCLC. DLL4-NOTCH signaling supports the maintenance of chemotherapy-resistant cancer stem cells and tumor vasculature, providing the rationale for targeting this pathway. In this study, forty-six treatment-naive patients received demcizumab, a humanized IgG2 monoclonal antibody against DLL4, together with standard chemotherapy to determine its maximum tolerated dose, safety, immunogenicity, preliminary efficacy, pharmacokinetics, and pharmacodynamics. This study has identified a truncated dosing regimen and recommended a phase II dose of demcizumab for subsequent clinical evaluation in combination with standard carboplatin and pemetrexed chemotherapy [154].

### 3.3. The Use of GSIs and Other Combined Therapies Has Contributed to Elucidating the Role of NOTCH Signaling in Gastric Cancer (GC) In Vitro and in Animal Models

Gastric carcinoma is one of the most common malignancies and a lethal cancer in the world. Hepatocyte growth factor (HGF) is clinically associated with gastric cancer progression and metastasis [155]. The NOTCH signaling pathway has also been firmly established as a key regulator of gastric stem cell proliferation, differentiation, and maintenance, acting both as an oncogene and a tumor suppressor gene [156]. Moreover, it plays a role in glandular fission, a process in which a glandular unit is divided into two due to elevated cell proliferation, thereby contributing to tumor development.

NOTCH signaling has been implicated in the initiation of oncogenesis through crosstalk with other pathways. NOTCH signaling and transcription factors STAT3 (signal transducer and activator of transcription 3) and Twist regulate tumor development and are critical regulators of gastric cancer progression. Twist and phosphorylated STAT3 levels were promoted by the activated NOTCH1 receptor in human stomach adenocarcinoma SC-M1. Furthermore, NOTCH1 and NOTCH ligand Jagged1 expressions were significantly associated with phosphorylated STAT3 and Twist levels in gastric cancer tissues of patients [157].

Patients with c-MET-positive tumors exhibit significantly reduced overall survival compared with those lacking c-MET expression, and JAGGED1 levels show a strong positive correlation with c-MET in human gastric cancer specimens. Stimulation with hepatocyte growth factor (HGF) increases JAGGED1 activity, which in turn induces a time-dependent upregulation of cyclooxygenase-2 (COX-2). These findings support the existence of a feed-forward loop between HGF/c-MET signaling and JAGGED1/NOTCH1 activation, which drives COX-2 expression and may contribute to therapeutic resistance [158]. The NOTCH2-COX-2 signaling axis also plays a key role in controlling gastric cancer progression. Constitutive expression of the NOTCH2 intracellular domain (NICD2), the activated form of the NOTCH2 receptor, promoted both cell proliferation and xenografted tumor growth of human stomach adenocarcinoma SC-M1 cells [159].

NOTCH signaling is associated with increased levels of CD44^+^ and CD133^+^ cells, which are recognized markers of gastric cancer stem cells (GCSCs) [66,160]. CD133^+^ gastric cancer stem cells (GCSCs) exhibited low RECK protein expression, a cysteine-rich protein with Kazal motifs known for its protease activity that inhibits metastasis and angiogenesis, and high NOTCH1 levels. RECK suppresses ADAM-mediated NOTCH1 activation. Moreover, by using the γ-secretase inhibitor DAPT, they demonstrated that NOTCH signaling was oncogenic in these tumors, with DAPT reducing the formation of GCSC-rich spheres by 25% compared to the control group [161]. CD44^+^ GCSCs, which showed elevated NOTCH1 expression and increased resistance to 5-fluorouracil (5-FU), were compared to CD44^−^ cells. Treatment with DAPT selectively affected CD44^+^ cells, leading to reduced self-renewal, diminished tumor initiation and migration, and enhanced sensitivity to 5-FU. In xenograft models, intraperitoneal administration of DAPT significantly inhibited tumor growth and epithelial–mesenchymal transition (EMT) [162].

Barat and colleagues also studied CD44^+^ cells, hypothesizing a functional link between NOTCH1 and the WNT/β-catenin pathway, another key signaling axis in gastric carcinoma. Treatment with GSI-IX produced dose- and time-dependent reductions in cell proliferation, migration, invasion, and tumor sphere size, along with increased apoptosis. Treated cells showed decreased levels of active NOTCH intracellular domain 1 (NICD1) and WNT signaling components. Similar results were observed in xenograft mice, with reduced tumor growth and increased necrosis in the GSI-IX-treated group [163].

NOTCH signaling and PTEN, a tumor suppressor gene frequently inactivated in gastric cancer (GC), seem to be related. Treatment of GC cells with GSI-I led to reduced tumor activity and increased PTEN expression compared to controls [164]. In xenograft models, the combination of GSI-I and paclitaxel resulted in significantly greater tumor growth inhibition than either agent as monotherapy. Lee and colleagues further evaluated GSI-I in GC cell lines and xenograft mice, confirming its efficacy and lack of significant side effects. When combined with 5-FU, both *in vitro* and *in vivo*, the dual treatment produced markedly greater tumor suppression than monotherapy [165].

Yao and colleagues observed that DAPT treatment increased ERK1/2 phosphorylation in GC cells, suggesting that NOTCH signaling may suppress this oncogenic kinase. Combining DAPT with PD98059, an ERK1/2 MAPK inhibitor, led to reduced tumor growth and increased apoptosis *in vitro*, with similar synergistic effects observed in xenograft models [166].

Kang et al. demonstrated that the concurrent administration of DAPT with anti-DLL4 enhances the anticancer and proapoptotic efficacy of the γ-secretase inhibitor in GC cell lines and xenograft mice. The combined treatment of DAPT with anti-DLL4 increased the expression of BAX and P53 while reducing the expression of Bcl-2. In contrast, GSI alone elevated only BAX and P53 levels, indicating that the downregulation of Bcl-2 plays a key role in the synergistic antitumor and proapoptotic effects observed with the combination therapy. This dual approach significantly enhanced apoptosis, reduced cell invasion, and decreased tumor size compared to control treatments [167].

### 3.4. GSIs Enhance the Efficacy of Targeted Therapies for Metastatic Melanoma in Preclinical Studies and Clinical Trials

Signaling through NOTCH receptors regulates cell proliferation and cell survival in several types of cancer, including malignant melanoma, with opposing results depending on the tissue context. Tumor progression/metastasis of malignant melanoma are complicated processes that require multiple cellular events, including cell proliferation, survival, migration, and invasion. NOTCH signaling appears to be a promising system for new therapeutic targets for the treatment of melanoma and perhaps the prevention of melanoma metastasis [168,169,170]. Stromal and cellular components of the tumor microenvironment strongly shape the molecular programs that drive melanoma growth and progression. Direct contact between melanoma cells and the distant epidermal layer activates NOTCH signaling and triggers vertical invasion. In the ligand-poor native microenvironment, MITF represses the miR-222/221 promoter in an RBPJκ-dependent manner. When radial growth brings melanoma cells into contact with differentiated keratinocytes expressing NOTCH ligands, NICD activation disrupts MITF binding to the miR-222/221 promoter, initiating invasion [171].

NOTCH3 is selectively upregulated in melanoma-endothelial co-cultures and is functionally linked to increased NOTCH pathway activity in melanoma cells. Induced NOTCH3 signaling enhances melanoma cell migration without increasing proliferation, and its expression is characteristic of malignant lesions but absent in benign nevi [172]. NOTCH signaling also promotes proliferation and invasion in uveal melanoma. Constitutively active NOTCH1 and NOTCH2 stimulate the growth of uveal melanoma cultures and can override the inhibitory effects of the γ-secretase inhibitor MRK003. MRK003 treatment reduces anchorage-independent clonogenic growth and invasion and decreases phosphorylation of STAT3 and ERK1/2 [173].

NOTCH signaling also appears to contribute to resistance against other targeted therapies. Krepler and colleagues combined the GSI RO4929097 with an ERK inhibitor (ERKi), finding that in ERKi-resistant cell lines, the combination significantly reduced cell viability and increased apoptosis compared to either agent as monotherapy. In xenograft models, the combination also produced superior tumor growth inhibition [174].

Approximately half of metastatic melanomas harbor mutations in the BRAF gene, for which targeted therapies using BRAF inhibitors (BRAFi) and MEK inhibitors (MEKi) are available. Zhu and colleagues explored the role of NOTCH signaling in acquired resistance to BRAFi. They found that combining DAPT with BRAFi reversed resistance in melanoma cell lines, suggesting that NOTCH signaling enables BRAFi-treated cells to escape senescence, a state that was re-induced by DAPT treatment [175]. Porcelli and colleagues tested the combination of MEKi and the GSI nirogacestat (PF-03084014), observing enhanced inhibition of cell proliferation and migration *in vitro* compared to monotherapy [176].

The combination of GSI-I with BCL-2 inhibitors (BCL2i) demonstrated greater efficacy than monotherapy in both melanoma cell lines and xenograft mice. The treatment increased apoptosis and reduced the population of ALDH^+^ MSCs [177].

FOXP3, a transcription factor associated with immune regulation and cancer, plays a critical role in melanoma progression. Its expression increases in metastatic melanoma cells under TGFβ stimulation, marking tumor aggressiveness and metastatic potential. Mechanistically, NOTCH1 was identified as the key driver of TGFβ-induced FOXP3 expression and blocking NOTCH1 with a GSI significantly reduced melanoma cell growth and survival. These findings highlight the importance of the TGFβ1/NOTCH1 signaling axis in regulating FOXP3 and advancing melanoma progression [178].

NOTCH signaling has also been implicated in the oncogenic potential of melanoma stem cells (MSCs). Kumar and colleagues associated CD133^+^ melanoma cells with MSCs and demonstrated, both *in vitro* and *in vivo*, enhanced proliferation, angiogenesis, epithelial–mesenchymal transition (EMT), metastasis, and chemoresistance. These cells exhibited overactivation of NOTCH1, and treatment with GSIs (GSI-IX and GSI-X) significantly reduced the number of CD133^+^ MSCs, leading to decreased migration and interaction with vascular endothelium [179].

In SK-MEL-2 metastatic melanoma cells, both membrane-bound DLK1 overexpression and soluble DLK1 treatment inhibit NOTCH receptor activation but paradoxically stimulate tumor cell proliferation. When membrane-bound and soluble DLK1 are combined, their inhibitory effects on NOTCH signaling are additive, leading to stronger NOTCH suppression and a reduction in tumor growth [76]. EGFL9/DLK2 behaves similarly: DLK2 overexpression suppresses NOTCH1 activation but unexpectedly enhances melanoma cell proliferation, whereas DLK2 downregulation activates NOTCH1 and inhibits proliferation. Furthermore, combining DLK2-overexpressing SK-MEL-2 cells with soluble DLK1 produces an even stronger NOTCH-inhibitory effect, resulting in further suppression of tumor growth [76]. These findings suggest that DLK1 and DLK2 exert context-dependent regulatory effects on NOTCH signaling and tumor behavior, highlighting their potential as therapeutic targets in specific cancer subtypes.

The contradictory effects of DLK1 or DLK2 proteins, non-canonical ligands of NOTCH receptors, observed in cancer may depend on several factors, particularly the level of DLK expression, which modulates their influence on NOTCH receptor activation and signaling, thereby determining their oncogenic or tumor-suppressor properties. Nueda and co-workers investigated the effects of DLK1 and DLK2 proteins, as monotherapy and in combination with the gamma-secretase complex inhibitor (GSI) DAPT, in SK-MEL-2 cells [76]. They demonstrated that the impact of DLK1 and DLK2 on oncogenic features depends on the expression levels, with tumor growth inhibition or activation achieved under different combinations despite being NOTCH inhibitors. However, it has been described that, depending on the concentration of DAPT used, opposite effects are obtained, being able to both inhibit and enhance the growth of metastatic melanoma cells [76]. Keyghobadi and colleagues observed that prolonged DAPT treatment also led to increased tumor growth *in vitro* and *in vivo* [180]. Nevertheless, a comprehensive and careful analysis is required to determine the optimal DLK1 and/or DLK2 peptides and GSIs dosages that achieve effective NOTCH receptor blockade that suppresses oncogenic features.

Among GSIs, RO4929097 is the only one to have entered clinical trials for metastatic melanoma. Tolcher and colleagues conducted a phase I trial in patients with metastatic or locally advanced tumors to determine the maximum tolerated dose (MTD), adverse effects, and preliminary efficacy. A total of 110 patients were enrolled and divided into three dosage groups (A, B, and C). Drug levels in all patients exceeded the threshold for antitumor activity. The most common adverse effects were gastrointestinal, with some cases of hypophosphatemia. Notably, one patient with melanoma showed a minor response, and 33% and 41% of patients in groups A and B, respectively, achieved disease stabilization [181].

Building on earlier findings, Lee and colleagues conducted a phase II clinical trial to assess the efficacy and tolerability of RO4929097 GSI in patients with metastatic melanoma. Among the 32 patients enrolled, most adverse effects were grade 1 or 2, with only six patients experiencing grade 3 toxicities, including hypophosphatemia. In terms of clinical response, one patient achieved a partial response lasting seven months and survived for over 28 months. Additionally, eight patients reached a stable disease state. However, overall efficacy was limited: the disease control rate at 12 weeks was 31%, the median progression-free survival (PFS) was 1.5 months, and the 6-month PFS rate was 9%. The 1-year survival rate was 50%, with a confidence interval of 23–66%. The authors attributed the modest outcomes to subtherapeutic drug levels [182].

More recently, Jayaprakash and colleagues revisited the use of RO4929097 in melanoma, identifying a potential synergistic effect at low doses when combined with radiotherapy (RT). *In vitro* studies also showed reduced cell migration with this combination [183].

### 3.5. Various Preclinical Studies and Clinical Trials Explore the Use of GSIs and Alternative Agents as Monotherapy and in Combination Therapies for Triple-Negative Breast Cancer (TNBC)

Triple-negative breast cancer (TNBC) is the most aggressive and heterogeneous breast cancer subtype, characterized by high metastatic rates, poor prognosis, and frequent drug resistance. TNBC patients face high risks of recurrence and metastasis, and current therapeutic options remain limited [184,185]. Aberrant NOTCH activation has been implicated in breast cancer, and multiple studies have identified distinct NOTCH1 and NOTCH2 rearrangements in subsets of breast cancer patients and cell lines, particularly in TNBC. These rearrangements generate membrane-tethered truncated NOTCH1 proteins lacking the S2 cleavage site, making them solely dependent on γ-secretase activity, or even truncated cytoplasmic NICD forms of NOTCH2 that no longer require γ-secretase cleavage. Clinically, high NOTCH1 expression correlates with poor prognosis, whereas elevated NOTCH2 levels are associated with improved outcomes [186,187,188].

Functional NOTCH receptor mutations are significantly enriched in TNBC. Using a novel antibody to detect active NOTCH3, constitutive NOTCH3 signaling has been demonstrated in a panel of basal breast cancer cell lines and in more than one-third of basal tumors. A NOTCH3 antagonist antibody suppressed the growth of basal lines, whereas a NOTCH3 agonist enhanced their transformed phenotype *in vitro* and in xenograft models. These findings reveal a ligand-independent activation mechanism and indicate that constitutive NOTCH3 signaling drives oncogenic programs in a subset of basal breast cancers [189].

EGFL9 (DLK2) is expressed at higher levels in triple-negative breast cancer (TNBC) cell lines compared with non-TNBC lines and functions as a key promoter of metastasis. It is both necessary and sufficient to drive cell migration, invasion, and distant metastatic spread. Mechanistically, EGFL9/DLK2 binds to and activates cMET, triggering downstream cMET-dependent signaling. Both proteins co-localize at the plasma membrane and within mitochondria. EGFL9/DLK2 also modulates COX activity and alters cellular metabolism, shifting cells toward a Warburg-like metabolic phenotype. Importantly, dual inhibition of cMET and glycolysis reverses EGFL9-driven stemness, highlighting EGFL9/DLK2 as a promising therapeutic target to prevent metastatic progression in TNBC [85]. In triple-negative breast cancer, high DLK1 expression has been associated with reduced tumor progression, whereas low DLK1 levels correlate with increased tumor size *in vivo*. Conversely, low DLK2 expression enhances tumor aggressiveness, while high DLK2 levels have been shown to prevent tumor formation in nude mice [74,75].

Activating variants in the PEST region of NOTCH1 are associated with aggressive phenotypes in human cancers, including TNBC, suggesting that PEST domain variants increase proliferation and invasiveness and reduce overall survival. Mutations within and upstream of the PEST domains of NOTCH1, NOTCH2, and NOTCH3 (as described in TCGA) arise through multiple genetic mechanisms and impair PEST domain function. These mutations and amplifications frequently activate the NOTCH pathway in a ligand-independent manner.

Patient-derived xenograft (PDX) models harboring PEST domain mutations showed high sensitivity to the GSI PF-03084014 [190]. However, introducing activating NOTCH1 variants into non-tumorigenic breast epithelial cell lines (MCF10A and hTERT-IMEC) produced increased transformative properties compared with a non-transformative PEST domain variant, including enhanced proliferation, migration, anchorage-independent growth, and MAPK pathway activation. Contrary to earlier reports, these activating NOTCH1 variants did not exhibit sensitivity to GSI PF-03084014 nor resistance to chemotherapies. These findings demonstrate that transformative phenotypes are variant-specific and do not necessarily correlate with chemotherapy or GSI sensitivity [191].

Stoeck and colleagues explored the relationship between NOTCH gene mutations and sensitivity to the GSI MRK003 in TNBC models. They found that therapeutic response was more closely linked to levels of active NOTCH intracellular domain (NICD) than to the presence of NOTCH mutations. In xenograft mice with elevated NICD levels, MRK003 was more effective, and *in vitro*, its combination with paclitaxel showed significant antitumor activity [192].

The combined use of the GSI MK-0752 and the MET inhibitor (METi) SU11274 in two TNBC cell lines with differing NOTCH1 expression was also analyzed in other work. MK-0752 as monotherapy did not inhibit cell growth, whereas SU11274 was effective, and its efficacy was enhanced when combined with MK-0752. Interestingly, MK-0752 was more effective than SU11274 in blocking colony formation, and the combination yielded the strongest inhibitory effect [193].

MDA-MB-231 triple-negative breast cancer cells respond effectively to treatment with a γ-secretase inhibitor (GSI), both as monotherapy and when paired with doxorubicin *in vitro* and *in vivo*. The combination of GSI with doxorubicin was not only practical but also improved therapeutic outcomes, highlighting its potential as a promising approach for treating triple-negative breast cancer [194].

An innovative strategy was proposed by Paroni and colleagues [195]. All-trans retinoic acid (ATRA), an unconventional therapy for breast cancer, is often ineffective in TNBC. However, their study revealed a correlation between ATRA sensitivity and elevated levels of active NICD1 in certain TNBC subtypes. In various TNBC cell lines, the combination of DAPT and ATRA proved more effective in suppressing tumor growth than either agent as monotherapy. Comparable results were obtained in TNBC xenograft mice treated with PF-03084014 and ATRA.

Recent literature highlights suberoylanilide hydroxamic acid (SAHA), a histone deacetylase inhibitor, as a promising therapeutic candidate for TNBC. SAHA may promote epithelial–mesenchymal transition (EMT), potentially due to NOTCH pathway overactivation. They combined SAHA with the GSI LY411575 in TNBC cell lines. This combination enhanced apoptosis, increased reactive oxygen species, induced mitochondrial depolarization, reduced EMT marker expression, and diminished breast cancer stem cell (BCSC) characteristics [196]. Sen and colleagues also used a multi-targeting TACE/ADAM17 and gamma-secretase of the NOTCH signaling pathway in TNBC via a drug repurposing approach using lomitapide [197].

The limited success of GSIs in clinical trials is largely attributed to their intestinal toxicity and potential immunological side effects, given the critical role of NOTCH signaling in T-cell activation, including CD8+ T cells within tumors. To overcome these limitations, Hossain and colleagues explored alternative agents that lack systemic toxicity and preserve tumor immunity [198]. They identified sulindac sulfide (SS), the active metabolite of the FDA-approved NSAID sulindac, as a promising GSI substitute. SS significantly inhibited nanosphere formation across human and murine TNBC models *in vivo*, *in vitro*, and *ex vivo*. In a transplantable TNBC mouse model (C0321), SS demonstrated potent single-agent antitumor activity and effectively suppressed NOTCH1 protein expression in tumors [198].

CB-103, an orally available pan-NOTCH inhibitor, is a promising and safer alternative to traditional γ-secretase inhibitors (GSIs) in breast cancer therapy. Unlike GSIs, CB-103 avoids gastrointestinal side effects due to its distinct mechanism of action. Preclinical evidence shows strong potential in luminal endocrine-resistant and triple-negative breast cancers (TNBCs) with confirmed NOTCH activity. When combined with SERDs or CDK inhibitors in endocrine-resistant recurrent breast cancers and with taxane-based chemotherapy in TNBC, CB-103 produced synergistic effects, boosting paclitaxel’s impact in TNBC-resistant xenograft models [199].

Through a genome-wide CRISPR-Cas9 screen, researchers found that SOX2 and NOTCH signaling form a reciprocal feedback loop: NOTCH signaling suppresses SOX2 via HEY target genes, while SOX2 inhibits NOTCH signaling by interacting with RBPJκ. This interplay creates distinct TNBC cell states: NOTCH-active cells with epithelial-like traits versus SOX2-driven cells with EMT features, stem cell properties, and drug resistance. Moreover, paclitaxel synergizes with the γ-secretase inhibitor crenigacestat (LY3039478), leading to tumor growth and metastasis reduction in NOTCH1^High^/SOX2^Low^ TNBC xenografts, while the synergistic combination of paclitaxel and dasatinib is efficient in NOTCH1^Low^/SOX2^High^ TNBC xenografts [200].

Growing evidence indicates that cancer originates from cancer stem cells (CSCs), which can be identified through aldehyde dehydrogenase (ALDH) activity–based flow cytometry [201]. As in other cancer types, tumor stem cells play a critical role in the tumorigenesis of triple-negative breast cancer (TNBC). CSCs contribute to TNBC initiation, progression, and chemotherapy resistance [202,203,204].

NOTCH signaling is a central pathway supporting CSC survival in TNBC [205]. Treatment of TNBC with PI3K or mTORC1/2 inhibitors can generate drug-resistant, NOTCH-dependent cancer stem cells (CSCs). The NOTCH ligand JAGGED1 rapidly induces AKT phosphorylation in a NOTCH1-dependent but RBPJκ-independent manner that requires mTOR and IKKα signaling. JAGGED1 also enhances mitochondrial respiration and glycolytic activity through AKT- and IKK-dependent mechanisms, and NOTCH1 partially localizes to mitochondria in TNBC cells. Pharmacologic inhibition of NOTCH cleavage using the GSI PF-03084014, combined with either the AKT inhibitor MK-2206 or the NF-κB/IKK inhibitor Bay11-7082, suppresses secondary mammosphere formation in CD90^High^ and CD44^+^CD24^Low^ CSC populations, with similar findings in patient-derived TNBC models. Collectively, these results indicate that targeting the NOTCH–AKT–NF-κB signaling axis may be an effective strategy to eliminate CSCs in TNBC tumors expressing NOTCH1 and wild-type PTEN [206]. The canonical ligand DLL4 activates NOTCH signaling in TNBC, and γ-Secretase-mediated NOTCH signaling regulates BCSC proliferation, differentiation, and metastasis [207].

Azzam and colleagues identified two subpopulations in TNBC cell lines, CD44^+^CD24^Low^ (hereafter CD24^Low^) and CD44^+^CD24^−^ (hereafter CD24^−^), both exhibiting characteristics of breast cancer stem cells (BCSCs). Treatment with the GSI RO4929097 inhibited sphere formation in CD24^low^ cells and significantly slowed tumor growth and metastasis in xenograft models, effects not observed in CD24^−^ cells [208]. Treatment of BCSC lines with DAPT decreased cell proliferation, increased apoptosis, reduced invasion, and diminished sphere formation. In xenograft mice, DAPT delayed tumor onset and slowed subsequent tumor growth [209].

Although DAPT, a potent γ-secretase inhibitor, effectively suppresses NOTCH signaling, its clinical translation is limited by poor biodistribution and off-target toxicity. To enhance selective delivery, DAPT was encapsulated into solid lipid nanoparticles (SLNs) and functionalized with DLL4 and DR5 antibodies, generating DLL4-DR5-DAPT SLNs. *In vitro*, these dual-targeted nanoparticles exhibited efficient uptake, strong cytotoxic activity, and significant inhibition of migration and invasion through NOTCH1 downregulation. They also promoted apoptosis by increasing caspase-8 expression and suppressed epithelial–mesenchymal transition (EMT) by upregulating E-cadherin and decreasing vimentin. *In vivo*, DLL4-DR5-DAPT SLNs demonstrated preferential tumor accumulation, markedly reduced tumor growth, lowered overall tumor burden, and improved long-term survival. Together, these findings show that DLL4/DR5 dual-functionalization greatly enhances targeted DAPT delivery, simultaneously inhibiting NOTCH signaling and inducing apoptosis, and represents a promising strategy for more effective TNBC therapy [207,210].

Mamaeva and colleagues inhibited NOTCH activity in breast cancer stem cells using glucose-functionalized nanoparticles loaded with γ-secretase inhibitors. These drug-loaded particles, conjugated to targeting ligands, produced cell-specific inhibition of NOTCH signaling *in vitro* and demonstrated enhanced tumor retention, resulting in significantly improved NOTCH inhibition and therapeutic outcomes *in vivo*. Oral administration of GSI DAPT-MSNPs regulated NOTCH activity in intestinal stem cells, further supporting the *in vivo* suitability of MSNPs for DAPT delivery. After systemic administration, MSNPs showed tumor accumulation and effective targeting. They were biocompatible, and particles not retained within tumors were degraded and eliminated primarily through renal excretion [211].

An innovative nanoparticle-based approach to counter resistance in cancers driven by NOTCH-EGFR interactions has been developed. Researchers developed nanoparticles carrying the γ-secretase inhibitor DAPT (NP-EB/DAPT) to block NOTCH signaling and enhanced tumor targeting with a specially engineered peptide (CF). This peptide links CREKA, which homes to tumors, with F3, a cell-penetrating peptide, via a pH-sensitive bond that prevents off-target activity. In the acidic tumor environment, the bond is cleaved, activating F3 to promote nanoparticle penetration into gastric cancer cells [212].

Schott and colleagues were the first to evaluate a GSI as a therapeutic agent for TNBC in humans. They studied chemotherapy-resistant BCSCs models treated with MK-0752 and found that, while tumors formed in 50% of control mice, none developed in the treated group. These findings led to a phase I clinical trial combining MK-0752 with docetaxel. Among 24 patients, one experienced grade 5 pneumonitis (likely due to docetaxel), while 11 had partial responses, 9 achieved stable disease, and 3 showed progression. Serial biopsies from six patients revealed a reduction in BCSCs populations [213].

A phase I trial combining the GSI PF-03084014 with docetaxel in 29 women with TNBC was also conducted. The study aimed to determine the maximum tolerated dose (MTD). Severe adverse events were reported, including one death from septic shock following febrile neutropenia. Grade 4 neutropenia occurred in 24 of the 29 patients, and hypophosphatemia was also observed. Treatment efficacy was limited [214].

In another phase I study, the MTD of RO4929097 in combination with paclitaxel and carboplatin in 14 patients with triple-negative breast cancer (TNBC) was analyzed. Like the previous study, several grade 4 adverse events were reported, the most significant being neutropenia and thrombocytopenia. In terms of clinical response, 5 patients showed a partial response, 4 achieved disease stabilization, and 5 exhibited residual disease [215]. RO4929097 has also been investigated in a phase Ib clinical trial for metastatic estrogen receptor-positive breast cancer (EPBCm). Means-Powell and colleagues administered RO4929097 alongside exemestane, an aromatase inhibitor, to 15 patients with EPBCm [216]. One dose-limiting grade 4 adverse event was observed, and grade 3 hypophosphatemia occurred in 13% of patients. Regarding efficacy, 7 patients demonstrated a partial response, and 7 maintained stable disease among the 14 evaluated.

The following two tables summarize the most relevant data reviewed in this work (Table 1 and Table 2).

## 4. Discussion

The use of γ-secretase inhibitors *in vitro* and in mouse xenograft models has emerged as a promising therapeutic strategy. These preclinical studies have shown encouraging results, particularly when GSIs are combined with other treatments. GSIs have demonstrated efficacy in enhancing the effects of various chemotherapeutic agents, for example, gemcitabine in pancreatic ductal adenocarcinoma (PDAC) [68,109], paclitaxel, osimertinib, erlotinib, crizotinib, and ABT-737 in non-small-cell lung cancer (NSCLC) [137,139,142,143,144,145,146,183], 5-FU in gastric cancer (GC) [164] or TNBC [200,212], and other inhibitors in TNBC [190,191,206]. Additionally, these combinations with radiotherapy (RT) have shown effectiveness in NSCLC and metastatic melanoma [137,143,183]. Research has also focused on the role of NOTCH signaling in cancer stem cells and its inhibition to overcome resistance to therapy and improve prognosis. A significant relationship has been identified between NOTCH signaling and gastric cancer stem cells (GCSCs), NSCLC, metastatic melanoma, and TNBC, suggesting promising therapeutic applications [127,134,140,160,161,162,163,177,179,208,209]. Another key area of investigation is the potential of GSIs to block epithelial–mesenchymal transition (EMT), a process implicated in GC, metastatic melanoma, and TNBC [162,179,196,217].

Recent studies have increasingly focused on combining GSIs with innovative therapeutic agents, yielding promising results. For instance, in TNBC, GSIs have been successfully combined with SAHA, ATRA, TACE inhibitor antibodies, and other technologies [124,189,195,196,197,198,199,207,210,211,212]. The combination of TACE/ADAM inhibitors and γ-secretase inhibitors could logically be more potent than treatment with GSIs alone, possibly with more severe and powerful side effects. Moreover, since lomitapide also inhibits these proteases, the synergistic effect of TACE and γ-secretase inhibitors together with lomitapide would be even greater. For this reason, the combination of SAHA, which inhibits cell-cycle-related genes, and the GSI LY411575, at appropriate doses, could be more suitable for a clinical trial. Table 1 classifies the data analyzed by GSI used, cancer type, study model (*in vitro* or xenograft), and outcomes. DAPT was the most frequently used GSI, followed by RO4929097.

GSIs have also entered clinical trials, although results remain limited (Table 2). Despite the promising preclinical data, clinical trials have yet to meet expectations. In a phase I trial involving stage IV PDAC patients, combining GSI with gemcitabine did not yield superior outcomes compared to gemcitabine as monotherapy [122]. Another phase II trial in metastatic PDAC was discontinued due to the discontinuation of the GSI under investigation [123]. In metastatic melanoma, a phase I trial of GSI monotherapy showed promising results, prompting a phase II trial. However, the latter produced limited outcomes, likely due to subtherapeutic dosing. Overall tolerability was acceptable, although severe hypophosphatemia was reported as a significant adverse event [181,182].

Although high DLL4 expression is significantly associated with poor prognosis in PDAC, NSCLC, and gastric cancer, and the *in vitro* and animal model results are promising, adjuvant therapy targeting DLL4-NOTCH signaling has many secondary effects, such as pulmonary hypertension and congestive heart failure after eight or more infusions. The combination of demcizumab with gemcitabine and other drugs currently used for these types of cancer is being investigated in several clinical studies at distinct phases in NSCLC and PDAC. These studies aim to evaluate the safety and efficacy of combining these agents compared with standard treatments and with demcizumab alone.

TNBC has been the cancer type with the highest number of clinical trials involving GSIs (Table 2). Three phase I trials evaluated different GSIs in combination with various chemotherapeutic agents, revealing several severe hematological and infectious adverse reactions. Although partial responses were observed in some patients, overall clinical efficacy was limited. A GSI was also evaluated in EPBCm with comparable results [174,208,213,214,215]. Table 1 presents a classification of clinical trial data by cancer type and outcomes, showing RO4929097 as the most frequently tested GSI.

A phase Ib trial evaluated the anti-cancer stem cell DLL4-binding agent demcizumab in combination with pemetrexed and carboplatin as first-line therapy for metastatic non-squamous NSCLC (Table 2). In this study, forty-six treatment-naive patients received demcizumab, a humanized IgG2 monoclonal antibody against DLL4, together with standard chemotherapy to determine its maximum tolerated dose, safety, immunogenicity, preliminary efficacy, pharmacokinetics, and pharmacodynamics [154].

Despite promising preclinical findings, several clinical studies of NOTCH/γ-secretase inhibitors have shown limited benefit in local tumor control and were discontinued. A major limitation has been gastrointestinal toxicity observed in preclinical models, a known consequence of NOTCH blockade [101]. This toxicity can be reduced with glucocorticoids or intermittent dosing schedules [218,219].

The varying degree of effectiveness of different GSIs across tumoral cells and cancer stem cells (CSCs) in preclinical studies and clinical trials from distinct tumor types is an intriguing and clinically relevant observation. Several factors may explain these discrepancies. One possible explanation is the reliance on cell lines with overactivated NOTCH signaling, which may not accurately represent the heterogeneity of human tumors. These cell lines were treated with GSIs and used to generate xenograft models in several cases. However, human tumors consist of diverse cell populations with varying levels of NOTCH receptor expression and activation. A specific GSI may only affect certain cell types, and its efficacy may depend more on activation levels than expression levels.

NOTCH signaling is highly context-dependent, and the relative contribution of each NOTCH receptor (NOTCH1-4) to tumor cells and CSC maintenance differs substantially between tumor types. Because GSIs inhibit the γ-secretase complex globally, their effectiveness depends on which NOTCH receptor is oncogenic in each cancer. A GSI may therefore be highly effective in tumors where the dominant oncogenic receptor is γ-secretase-dependent but less effective in settings where alternative NOTCH receptors or parallel pathways sustain tumoral and CSC function. Many studies have not accounted for the specificity of GSIs toward different NOTCH receptors. This non-selective inhibition may lead to adverse effects, such as increased incidence of non-melanoma skin neoplasms. For example, indiscriminate inhibition of all NOTCH receptors may suppress tumor-suppressive pathways, as seen with NOTCH2 in breast cancer [59]. Additionally, low levels of NOTCH inhibition by DLK1 and DLK2 proteins may paradoxically increase cell proliferation, as observed by Nueda and colleagues, Naranjo and Colleagues and others [74,75,76].

The γ-secretase complex itself is heterogeneous. Its composition (e.g., PSEN1/PSEN2, APH1A/APH1B isoforms) varies across tissues and tumor types, influencing substrate specificity and drug sensitivity. This biochemical diversity means that a particular GSI may inhibit γ-secretase efficiently in one cellular context but not in another.

Tumoral cells and CSCs rely on multiple compensatory pathways (such as WNT, Hedgehog, PI3K/AKT, and NF-κB). In some cancers, these pathways can partially or fully bypass NOTCH inhibition, reducing the apparent effectiveness of GSIs even when NOTCH is aberrantly activated.

Pharmacokinetic and pharmacodynamic differences, including drug uptake, efflux pump activity, and intracellular metabolism, vary across tumor cells and CSCs from different tumors and can limit the intracellular concentration of GSIs, thereby modulating their inhibitory capacity. GSIs themselves differ in potency, selectivity, and off-target effects, which can contribute to variable responses across tumor cells and CSC types.

Finally, ligand-independent NOTCH activity is an important consideration in cancer because it opens the possibility of using alternative inhibitors with potentially fewer adverse effects. Several mechanisms can drive ligand-independent NOTCH signaling, including mutations that remove key processing sites or modified PEST domains, aberrant receptor overexpression that promotes spontaneous activation, and activation events occurring during endocytic or lysosomal trafficking [220].

To enhance the therapeutic index of γ-secretase inhibitors in clinical trials, combination strategies and optimized delivery systems should be further explored. Nanoparticle-based approaches are particularly promising [221]. For example, pH-responsive peptide-functionalized nanoparticles co-delivering erlotinib and DAPT effectively suppressed TNBC progression [212], and DT7-modified lecithin nanoparticles carrying a GSI plus dexamethasone inhibited T-cell acute lymphoblastic leukemia while reducing gastrointestinal toxicity [222].

On the other hand, novel approaches to KRAS inhibition are advancing rapidly, and the potential for stage-dependent synergy between γ-secretase inhibitors (GSIs) and KRAS inhibitors is particularly relevant for malignancies such as NSCLC and PDAC. ALK, a member of the insulin-receptor superfamily, regulates cellular growth, proliferation, and survival. ROS1, which is highly homologous to ALK, performs similar physiological functions. Overexpression of either kinase is strongly associated with tumor development and metastasis, making both ALK and ROS1 important therapeutic targets in NSCLC. Multiple ALK inhibitors have demonstrated substantial clinical benefit in ALK^-^ or ROS1^+^ NSCLC. However, despite initial responses to tyrosine kinase inhibitors, patients almost invariably develop resistance, ultimately leading to treatment failure [223,224].

Patients with peripheral T-cell lymphomas (PTL), including anaplastic large cell lymphoma (ALCL), continue to receive intensive multi-agent chemotherapy, which is highly toxic and often followed by relapse. PTL tumor cells show strong NOTCH1 expression [225]. In fact, the same research group performed immunohistochemical analysis of NOTCH1 in lymph nodes from PTL-NOS and systemic ALCL (both ALK^+^ and ALK^–^) and found comparable expression patterns across all three subtypes. In the ALK^+^ ALCL cell line Karpas-299, pharmacologic blockade of NOTCH signaling with GSI I proved significantly more effective than GSI IX, XX, or XXI in reducing cell viability and inducing apoptosis. Nevertheless, inhibiting the NOTCH pathway is associated with cytotoxicity [226].

Conversely, Larose and colleagues conducted whole-exome sequencing of ALK^+^ ALCL and identified NOTCH1 mutations that promote cellular growth. They also showed that targeting NOTCH1, either with γ-secretase inhibitors or by shRNA-mediated silencing, induces apoptosis. Moreover, combining NOTCH1 inhibition with the ALK inhibitor crizotinib produced additive or synergistic antitumor effects *in vitro*. These findings suggest that such combination therapy could be a promising strategy to overcome ALK-inhibitor resistance in various cancers while potentially reducing adverse effects [227].

Synergistic effects seen in some combination therapies involving NOTCH inhibition indicate that lower doses of each agent may achieve comparable efficacy while reducing normal-tissue toxicity. These dose reductions could allow longer treatment courses and potentially improve survival. Combination therapies show promise but can promote stem-like cells and EMT. GSIs counteract chemotherapy resistance by suppressing EMT, stem-like transformation, and survival signaling [228,229].

γ-Secretase Inhibitors (GSIs) also act as anti-angiogenic agents, blocking endothelial growth and tumor vascularization in cancer models [150,230]. Because NOTCH inhibition also remodels tumor vasculature [231,232], combinations with radiation or chemotherapy require careful timing. In clinical practice, concurrent polychemotherapy with fractionated radiotherapy improves survival in locally advanced disease. Follow-up studies should therefore determine whether adding NOTCH inhibition to chemotherapy provides superior tumor-growth delay compared with current polychemotherapy regimens when both are paired with fractionated radiotherapy. A noncanonical, pro-apoptotic role of NOTCH3 in tumor angiogenesis has also been identified. In this setting, aberrant NOTCH3 expression induces endothelial cell death, while the ligand Jagged-1, upregulated in a subset of human cancers, suppresses this apoptosis. Studies using NOTCH3-deficient mice show that silencing NOTCH3 in the stromal compartment enhances tumor growth and angiogenesis. These findings indicate that part of the antitumor effect observed with γ-secretase inhibition arises from NOTCH3-mediated apoptosis in endothelial cells [233].

The tumor microenvironment may generate resistance to drugs, such as cancer-associated fibroblasts (CAFs) in pancreatic cancer, which are highly chemoresistant, forming protective structures and secreting IL-6. Biomarkers like IL6/IL8 predict resistance to GSIs (e.g., RO4929097), guiding patient selection for better outcomes [120]. There are some clinical advances, such as those performed with Nirogacestat (PF-03084014), which shows striking benefit in desmoid tumors (71.4% response rate, durable for >6 years). Other GSIs (e.g., AL-102) are in late-stage trials, expanding potential beyond desmoid tumors to multiple myeloma [234,235,236]. Preclinical work with GSI RO4929097 showed that tumor cell lines with high IL6 and IL8 expression were resistant, as the drug no longer impacted angiogenesis or fibroblast infiltration. Xenograft models confirmed that IL-6/IL-8 overexpression predicts *in vivo* resistance. Clinically, Phase I data revealed that patients with low baseline IL6 and IL8 levels were more likely to benefit from treatment. [237].

CRISPR is reshaping oncology by enabling precise gene editing for modeling tumors, immune responses, and drug resistance. It identifies oncogenesis and chemoresistance mechanisms, offering new therapeutic opportunities beyond conventional NOTCH inhibition. CRISPR advances immune-based treatments like CAR T cells and direct antitumor agents with bispecific antibodies [238,239,240]. BCMA-targeted therapies (CAR T cells, bispecific antibodies) revolutionize multiple myeloma treatment but face relapse due to resistance mechanisms [241,242]. Finally, innovative trials combine immunotherapy (pembrolizumab) with carbon ion radiotherapy, aiming to enhance immune responses while sparing healthy cells. Combination therapies and precision strategies are key to long-term cancer control [243].

## 5. Conclusions

NOTCH signaling plays a key role in the five types of tumors under study and in cancer stem cells. γ-secretase inhibitors (GSIs) show promise *in vitro* and in mouse xenograft models and enhance the effects of chemotherapy and radiotherapy. GSIs can block epithelial–mesenchymal transition (EMT) and may help overcome therapy resistance and improve prognosis. However, clinical trial results remain limited despite preclinical promise. Future research should identify oncogenic NOTCH receptors/ligands to enable receptor-specific GSIs or alternative inhibitors (e.g., CB103, ADAM inhibitors, DLK proteins, and other NOTCH regulators), combined with patient stratification based on NOTCH activation levels. Lower-dose combinations show promise but may induce stem-like traits and EMT, requiring careful monitoring. Trials are integrating new strategies such as immunotherapy with CRISPR, CAR T cells, and bispecific antibodies to model tumors and drug resistance more precisely. Studying immune evasion, investigating microenvironment-driven resistance, and targeting angiogenesis remain critical strategies. Finally, leveraging big data and AI can enable individualized treatments, including male- and female-specific considerations.

A formal risk-of-bias assessment was not conducted in this review. Although structured tools such as ROBINS-I or OHAT are recommended for systematic reviews intended to inform guideline development or support causal inference, the present review had a different purpose. Our aim was to map and describe the available evidence across heterogeneous study designs and exposure contexts, rather than to derive pooled effect estimates or formally grade the certainty of the evidence.

## Figures and Tables

**Table 1:** Summary of the effect of different GSIs and some other alternatives *in vitro* studies and xenograft mice in the cancer types under study in this review. PDAC: pancreatic ductal adenocarcinoma. TNBC: triple-negative breast cancer. GC: gastric carcinoma. NSCLC: non-small-cell lung cancer.

GSI	Type of Cancer	Type of Study	Main Results	Reference
MRK003	PDAC	*In vivo* (xenograft) ± gemcitabine	The combination blocked tumor progression	[68,118]
	NSCLC	*In vivo +* erlotinib	Induced cell death in hypoxic tumors and decreased metastasis to the liver and brain. Prolonged median survival in mice	[146]
		*In vitro* and *in vivo*	Reduces the clonogenic potential of cancer cell lines, and this effect can be reversed by expressing a constitutively active form of NOTCH3. *In vivo*, there is no clear impact on tumorigenicity	[147]
		*In vitro* and *in vivo*	GFP reporter to identify a subset of cells with high NOTCH activity that formed more tumorspheres in serum-free conditions, were resistant to chemotherapy, and remained tumorigenic in serial xenotransplantation assays, which failed to regenerate tumors after reimplantation into mice	[148]
		Mesenchymal cells and 9 treatment-naïve patients	Reduced collagen production and suppressed invasive behavior	[149]
	Metastatic melanoma	Primary tumor samples, cell lines, and xenograft mouse model	Reduces anchorage-independent clonogenic growth and invasion and decreases phosphorylation of STAT3 and ERK1/2	[173]
	TNBC	*In vitro* and *in vivo* + placitaxel	Greater antitumor activity of the combination in cells with higher NICD levels	[192]
GSI-IX	PDAC	*In vivo* (xenograft) + AG-490	Mice treated with the combination showed no visible tumors	[119]
		*In vitro* and in a xenograft mouse model	Reduced the growth of pancreatic tumor-initiating CD44^+^/EpCAM^+^ cells	[98]
	GC	*In vitro*, in CD44^+^ cells	Smaller tumor spheres and increased apoptosis	[163]
		*In vivo* (xenograft mouse model)	Reduced tumor growth and increased necrosis	
	NSCLC	*In vitro* + paclitaxel	Synergistic antitumor effect by modulating the intrinsic apoptosis pathway and enhancing cell death. Reduced NOTCH3–induced chemoresistance in a concentration-dependent manner	[145]
	Metastatic melanoma	*In vitro*	GSI decreased CD133^+^ cells (MSCs)	[179]
GSI-X				
PF-03084014 (Nirogacestat)	PDAC	*In vivo* (xenograft) ± gemcitabine	Only in combination did it show antiproliferative activity and reduce cancer stem cells	[109]
	Metastatic melanoma	*In vitro* + MEKi	The combination was more effective in stopping proliferation and migration	[176]
	TNBC	*In vitro* and patient-derived xenograft (PDX) models *+* AKT inhibitor MK-2206 or the IKK-targeted NF-κB inhibitor Bay11-7082	High sensitivity in patients harboring PEST domain mutations. Activating NOTCH1 variants did not exhibit sensitivity to this GSI nor resistance to chemotherapies	[190,191]
			Suppresses secondary mammosphere formation from sorted CD90^High^ or CD44^+^CD24^Low^ CSCs	[206]
DAPT	PDAC	*In vitro*	CAF monocultures hardly responded to DAPT, which suggested that CAFs are more resistant to standard chemo treatments than the epithelial cancer cells. Elevated levels of IL-6 were also associated with a reduced response to therapy	[120]
	NSCLC	*In vitro* and *in vivo* (xenograft)	Treatment with DAPT markedly decreases primary pulmospheres in CD24^+^ITGB4^+^NOTCH^High^ cells	[134]
		*In vitro* + cisplatin	Decrease in the appearance of CD133^+^, ALDH^+^ LCSC cells, with lower resistance to cisplatin	[140]
		KRASG12V-driven NSCLC. *In vivo*	GSI treatment upregulated DUSP1, leading to reduced phospho-ERK levels	[133]
		*In vitro* and lung adenocarcinoma tumors that were xenotransplanted into nude mice.	Reduced endothelial cell proliferation, suppressed the formation of capillary structures, opposed the sprouting of microvessel outgrowths, and potently inhibited the growth and vascularization	[150]
	GC	*In vitro*	Inhibited the formation of GCSC-rich spheres by 25%	[161]
		*In vitro*, in CD44^+^ and CD44^-^ cells	CD44^+^ cells, behaving as GCSCs, showed a greater antitumor response to GSI. Enhanced sensitivity to 5-FU	[162]
		*In vivo* (xenograft)	Significant inhibition of tumor growth and EMT	
		*In vitro* ± PD98059 *In vivo* (xenograft) ± D98059	Reduced tumor growth and increased apoptosis in combination	[166]
		*In vitro* ± anti-DLL4	Significant increase in apoptosis and reduced invasion and tumor size.	[167]
	Metastatic melanoma	*In vivo* (xenograft) ± BRAFi	Reversal of melanoma cell resistance to BRAFi	[175]
		*In vitro* ± DLK1 and/or DLK2 levels	Dose-dependent effect of DAPT: decreased proliferation at high doses, increased at low doses. The combination reduced cell proliferation	[76]
		*In vitro* and *in vivo* (xenograft)	Long-term use of DAPT increased tumor growth	[180]
	TNBC	*In vitro,* in BCSCs	Reduced proliferation and increased apoptosis	[209]
		*In vivo* (xenograft)	Delay in tumor formation and reduced subsequent growth	
		Nanoparticles carrying DAPT. *In vivo* (xenograft) + erlotinib + director peptide	The nanoparticle reduced tumor growth and cell migration	[212]
		Lipid nanoparticles (SLNs) carrying DAPT and functionalized with DLL4 and DR5 antibodies. *In vitro* and *in vivo*	*In vitro*, efficient uptake, strong cytotoxic activity and apoptosis, and significant inhibition of EMT and migration-invasion. *In vivo*, tumor accumulation markedly reduced tumor growth, lowered overall tumor burden, and improved long-term survival	[207,210]
		Glucose-functionalized nanoparticles loaded with DAPT, conjugated to targeting ligands. *In vitro* and *in vivo*	Cell-specific inhibition of NOTCH signaling *in vitro* and demonstrated enhanced tumor retention *in vivo*. Oral administration regulated NOTCH activity in intestinal stem cells	[211]
		*In vitro* + ATRA	The combination was more effective in inhibiting tumor growth	[195]
GSI-34	NSCLC	*In vivo* (xenograft) with CD166^+^Lin^-^ LCSCs ± cisplatin	CD166^+^Lin^−^ showed intrinsic resistance to cisplatin, which reversed with GSI. The combination effectively reduced tumor size	[127]
BMS-708163	NSCLC	*In vitro,* in NSCLC-gefitinib- resistant cells + gefitinib	High doses of GSI reversed resistance to gefitinib and formed smaller colonies	[141]
		*In vivo* (xenograft) + gefitinib	The combination produced considerable inhibition of tumor growth	
BMS-906024		*In vitro*, in NSCLC cells + RT ± paclitaxel and crizotinib	Monotherapy + RT did not show significant reduction. It was observed with the combinations. Also, with DBZ	[137]
		*In vivo* (xenograft) + paclitaxel	The combination enhanced the cytotoxic effect of paclitaxel	[138,139]
GSI-XX		*In vivo* (xenograft) + RT	The combination caused a significant delay in tumor growth	[143]
		*In vitro* and *in vivo* experiments with osimertinib	Impaired drug-tolerant persistence, suppressed phospho-ERK, and enhanced DUSP1 expression	[142]
		*In vitro* and *in vivo* + ABT-737	Treatment with either agent and in combination inhibited cell proliferation in a dose-dependent manner and regulated the expression of apoptosis proteins	[144]
GSI-I		*In vitro* + RT	Higher level of apoptosis than isolated RT	[143]
	GC	*In vitro* and *in vivo* (xenograft) + paclitaxel or 5-FU	Increased activity of PTEN, a tumor suppressor gene Both combinations were more effective than monotherapy	[164,165]
	Metastatic melanoma	*In vivo* (xenograft) and *in vitro* + BCL2i	The combination was more effective than monotherapy	[177]
RO4929097		*In vitro* + ERKi	Sensitization to ERKi in cell lines that did not respond to it in monotherapy	[174]
		*In vivo* (xenograft) + ERKi	The combination was more effective than monotherapy	
		*In vitro* + RT	Synergism at low doses in combination	[183]
	TNBC	*In vitro,* in CD24^Low^ and CD24^−^ (BCSCs) cells	Inhibition of CD24^Low^ sphere growth	[208]
		*In vivo* (xenograft) with CD24^Low^ and CD24^−^ (BCSCs) cells	Halted tumor growth and metastasis in CD24^Low^ models	
MK-0752		*In vitro.* Various levels of NOTCH expression + METi	The combination showed synergism in halting cell growth	[193]
LY411575		*In vitro* + SAHA	SAHA in monotherapy promoted EMT. The combination reduced EMT and increased apoptosis	[196]
LY3039478 (Crenigacestat)		TNBC xenografts + paclitaxel + dasatinib	Tumor growth and metastasis reduction	[200]
OTHERS (Evodiamine)	NSCLC	*In vitro*	Not a GSI but behaves like one. It reduced cell proliferation and metastasis	[153]
OTHERS (Exosomes)	PDAC	*In vitro*	Exosomes released by SOJ-6 pancreatic tumor cells induce ligand-independent NOTCH1 inactivation and promote cell death	[124]
OTHERS (NSAID sulindac (SS))	TNBC	*In vitro*, *in vivo*, and *ex vivo*	Significantly inhibited nanosphere growth in all human and murine TNBC models. Eliminated NOTCH1 protein expression in tumors	[198]
OTHERS (CB103, a pan-NOTCH inhibitor)		Endocrine-resistant BC xenografts	When combined with SERDs or CDK inhibitors in endocrine-resistant recurrent breast cancers and with taxane-based chemotherapy in TNBC, CB-103 produced synergistic effects, boosting paclitaxel’s impact	[199]
OTHERS (Lomitapide)		*In vitro*	Multi-targeting TACE/ADAM17 and gamma-secretase complex of the NOTCH signaling pathway	[197]
OTHERS (NOTCH3 Ab)		*In vitro*	Suppressed the growth of basal lines. ligand-independent activation mechanism	[189]

**Table 2:** Summary of the different clinical trials conducted with GSIs and some other alternatives in the cancer types under study in this review. PDAC: pancreatic ductal adenocarcinoma. TNBC: triple-negative breast cancer. NSCLC: non-small-cell lung cancer. EPBCm: metastatic estrogen receptor-positive breast cancer. Table created by the authors.

Type of Cancer	GSI	Phase	Results	Reference
PDAC	MK-0752 + gemcitabine	I	14 out of 44 patients reached a stable condition in both monotherapy and combination therapy. Gastrointestinal disorders and anemia were observed. ClinicalTrials.gov identifier: NCT01098344 (https://clinicaltrials.gov/study/NCT01098344) (accessed on 15 December 2025)	[122]
	RO4929097	II	The trial could not be completed because GSI synthesis was discontinued. Clinicaltrials.gov identifier: NCT01232829 (https://cdek.pharmacy.purdue.edu/trial/NCT01232829/) (accessed on 15 December 2025)	[123]
Metastatic melanoma	RO4929097	I	In two groups of 110 patients, 33% and 41% reached a stable condition. Hypophosphatemia was noted. Cancer Therapy Evaluation Program (CTEP)	[181]
		II	Of 32 evaluated patients, 1 had a partial response and 8 reached a stable condition. Hypophosphatemia was also observed. ClinicalTrials.gov identifier: NCT01120275 (https://clinicaltrials.gov/study/NCT01120275) (accessed on 15 December 2025)	[182]
TNBC	MK-0752 + docetaxel	I	Among 24 patients, 11 had a partial response, 9 reached a stable condition, and 3 showed tumor progression. There was one case of severe pneumonitis. ClinicalTrials.gov identifier: NCT00645333 (https://cdek.pharmacy.purdue.edu/trial/NCT00645333/) (accessed on 15 December 2025)	[213]
	PF-03084014 + docetaxel	I	29 women showed limited treatment efficacy, with severe hematologic and infectious reactions. ClinicalTrials.gov identifier: NCT01876251 (https://clinicaltrials.gov/study/NCT01876251) (accessed on 15 December 2025)	[214]
	RO4929097 + placitaxel + carboplatin	I	Of 14 evaluated patients, 5 had a partial response, 4 reached a stable condition, and 5 had residual disease. *Neutropenia* was reported (http://ctep.cancer.gov/protocol) (accessed on 15 December 2025)	[215]
NSCLC	pemetrexed and carboplatin + demcizumab (DLL4 Ab)	Ib	46 treatment-naive patients received demcizumab, a humanized DLL4 antibody, together with standard chemotherapy to determine its maximum tolerated dose, safety, immunogenicity, preliminary efficacy, pharmacokinetics, and pharmacodynamics. ClinicalTrials.gov identifier: NCT01189968 (https://clinicaltrials.gov/study/NCT01189968) (accessed on 15 February 2026)	[154]
EPBCm	RO4929097 + exemestane	Ib	Among 14 evaluated patients, 7 had a partial response and 7 reached a stable condition. ClinicalTrials.gov identifier: NCT01149356 (https://clinicaltrials.gov/study/NCT01149356) (accessed on 15 December 2025)	[216]

## Data Availability

No new data were created. All data analyzed in this study are included in this published systematic review [and its Supplementary Information Files]. This work will be deposited in the RUIdeRA institutional repository at University of Castilla-La Mancha, Spain.

## Supplementary Materials

The following supporting information can be downloaded at: https://www.mdpi.com/article/10.3390/cells15050468/s1, Figure S1: Structure of canonical ligands of the NOTCH receptors. The JAG1/2 ligands have almost twice as many EGF-like repeats as the DLL1, 3, and 4 ligands. They all possess the DSL domain which interacts with the EGF-like 11 and 12 repeats of the receptors. CRD (cysteine-rich domain). MNNL: Module at N-terminal Domain of NOTCH Ligands. DOS: (Delta and OSM-11).; Figure S2: Structure of the non-canonical NOTCH receptor ligands DLK1 and DLK2. Unlike canonical ligands, DLK1 and DLK2 lack the DSL domain but contain DOS (Delta and OSM-11) motifs in their amino-terminal regions. These motifs, also found in some canonical ligands, enable interaction with NOTCH receptors, allowing DLK1 and DLK2 to compete with canonical ligands and inhibit NOTCH signaling. DLK1 is further processed by ADAM17 (also known as TACE), generating soluble forms; Figure S3: Hallmarks of cancer influenced by the NOTCH signaling pathway. Oncogenic effects are highlighted in green, while tumor suppressor effects are shown in orange. The table also indicates the types of cancer in which each hallmark may be implicated. Abbreviations: CLL: chronic lymphocytic leukemia; ACC: adenoid cystic carcinoma; SCC: squamous cell carcinoma; Breast Ca: breast cancer; TICs: tumor-initiating cells. Table S1: PRISMA checklists.

## Abbreviations

The following abbreviations are used in this manuscript:

ABT-737	Bcl-2 inhibitor
ADAM	A Disintegrin And Metalloproteinase
ADM	Acinar-to-ductal metaplasia
AKT	Protein kinase B (PKB)
ALCL	Anaplastic large cell lymphoma
ALDH	Aldehyde dehydrogenase
ALK	Anaplastic lymphoma kinase
ATRA	All-trans retinoic acid
BAX	BCL-2-associated X protein
Bcl-2	B cell lymphoma-2
BCL2i	BLC-2 inhibitors
BCSCs	Breast cancer stem cells
BCMA	B-cell maturation antigen
BRAFi	BRAF inhibitor
BRCA1/2	Breast cancer gene 1/2
CAFs	Cancer-associated fibroblasts
CAR-T	Chimeric artificial T cell receptors
CB-103	Non-gamma-secretase inhibitor
CD44	Cell Surface Glycoprotein CD44
CD133	Transmembrane glycoprotein CD133
cMET	Mesenchymal-epithelial transition receptor tyrosine kinase
COX	Cyclooxygenase
CREKA	Pentapeptide lineal biologically active compound
CRISPR	Clustered regularly interspaced short palindromic repeats
CSCs	Cancer stem cells
CSL	CBF1/suppressor of hairless/LAG-1, also known as RBP-Jκ
DAPT	GSI-IX
DBZ	Dibenzazepine
DDR1	Discoidin domain receptor 1
DLK1	Delta like homolog 1
DLK2	Delta like homolog 2
DLL1	Canonical Delta-Like1 ligand
DLL3	Canonical Delta-Like3 ligand
DLL4	Canonical Delta-Like4 ligand
DOS	Delta and OSM-11 Motif
DR5	Death receptor 5
DSL	Delta/serrate/LAG-2 domain
DT	Desmoid tumors
DTP	Drug-tolerant persisted cells
DUSP1	Dual-specificity phosphatase 1
EGF	Epidermal growth factor
EGFR	Epidermal growth factor receptor
EGFL9	Epidermal growth factor like (DLK2)
EMT	Epithelial–mesenchymal transition
EPBCm	Estrogen receptor-positive metastatic breast cancer
EpCAM	Epithelial cell adhesion molecule
ErbB-4	EGFR subfamily of receptor tyrosine kinases
ERK	Extracellular signal-regulated kinase
ERKi	ERK MAPK inhibitor
EVO	Evodiamine
5-FU	5-fluorouracil
FOXP3	Forkhead box P3
GC	Gastric cancer
GCSCs	Gastric cancer stem cells
GFP	Green fluorescent protein
GSC	γ-secretase complex
GSI	γ-secretase inhibitor
HES1	Hairy and enhancer of split-1
HEY	Hes related family BHLH transcription factor with YRPW motif
HGF	Hepatocyte growth factor
IKK-β	Inhibitor of nuclear factor kappa-B kinase subunit beta
IL	Interleukin
IGF-1R	Receptor of growth factor similar to insulin 1
JAG1	Canonical Jagged 1 ligand
JAG2	Canonical Jagged 2 ligand
KRAS	Kirsten rat sarcoma
LCSCs	Lung cancer stem cells.
LFNG	Lunatic fringe
mAb	Monoclonal antibody
MAML	Mastermind-like protein
MAPK	Mitogen-activated protein kinase
MEK	Mitogen-activated protein kinase 1 (MAP2K1)
MEKi	MEK inhibitor
METi	MET inhibitor
MITF	Ligand-poor native microenvironment
MM	Multiple myeloma
MSNPS	Mesoporous silica nanoparticles
MTD	Maximum tolerated dose
mTOR	Mammalian target of rapamycin
MSC	Melanoma stem cells
NCOR1	Nuclear receptor corepressor 1
NECD	NOTCH extracellular domain
NF-κB	Nuclear factor enhancing kappa light chains of activated B cells
NICD	NOTCH intracellular domain
NP-EB/DAPT	Nanoparticles carrying the γ-secretase inhibitor
NRR	Negative regulatory region
NSCLC	Non-small-cell lung cancer
NUMB	Cell fate determinant
p53	Tumor suppressor protein 53
PanIN	Pancreatic intraepithelial neoplasia
PARP	Poly(ADP-ribose) polymerase
PDAC	Pancreatic ductal adenocarcinoma
PDX	Patient-derived xenografts
PEST	Proline, glutamic acid, serine, and threonine domain
PD-1	Programmed cell death protein 1
PFS	Progression-free survival
PI3K	Phosphoinositide 3-kinase
PTEN	Phosphatidylinositol-3,4,5-trisphosphate 3-phosphatase
PTL	Peripheral T-cell lymphomas
RBP-Jκ	Recombination signal binding protein for immunoglobulin kappa J region
RECK	Reversion-inducing cysteine-rich protein with Kazal motifs
ROS1	Proto-oncogene tyrosine-protein kinase ROS
RT	Radiotherapy
POGLUT-1	Endoplasmic reticulum protein O-glucosyltransferase (RUMI)
SAHA	Suberoylanilide hydroxamic acid
shRNA	Short hairpin RNA
SOX2	SRY-related HMG-box 2
SS	Sulindac sulfide
STAT3	Signal transducer and activator of transcription 3
TACE	Tumor necrosis factor (TNF)-converting enzyme
TGF-β	Transforming growth factor beta
TMD	Transmembrane domain
TNBC	Triple-negative breast cancer
TPCs	Subpopulation of tumor-propagating cells
Twist	Class A basic helix–loop–helix protein 38 (bHLHa38)
VEGF	Vascular endothelial growth factor
VEGFR1	Receptor of vascular endothelial growth factor 1
WNT	Wingless and Int-1
2D	Two dimensions
3D	Three dimensions
WHO	World health organization
