# Supplementary material for: Modulation of Oncogenic NOTCH Signaling in Highly Aggressive Malignancies by Targeting the γ-Secretase Complex: A Systematic Review
Source: Cells. 2026 Mar 5;15(5):468. doi: 10.3390/cells15050468 (PMC12984106; doi:10.3390/cells15050468)
Supplement: Supplementary file 1 [file cells-15-00468-s001.zip › cells-4087857-supplementary/cells-4087857-supplementary.pptx]

## Slide 1
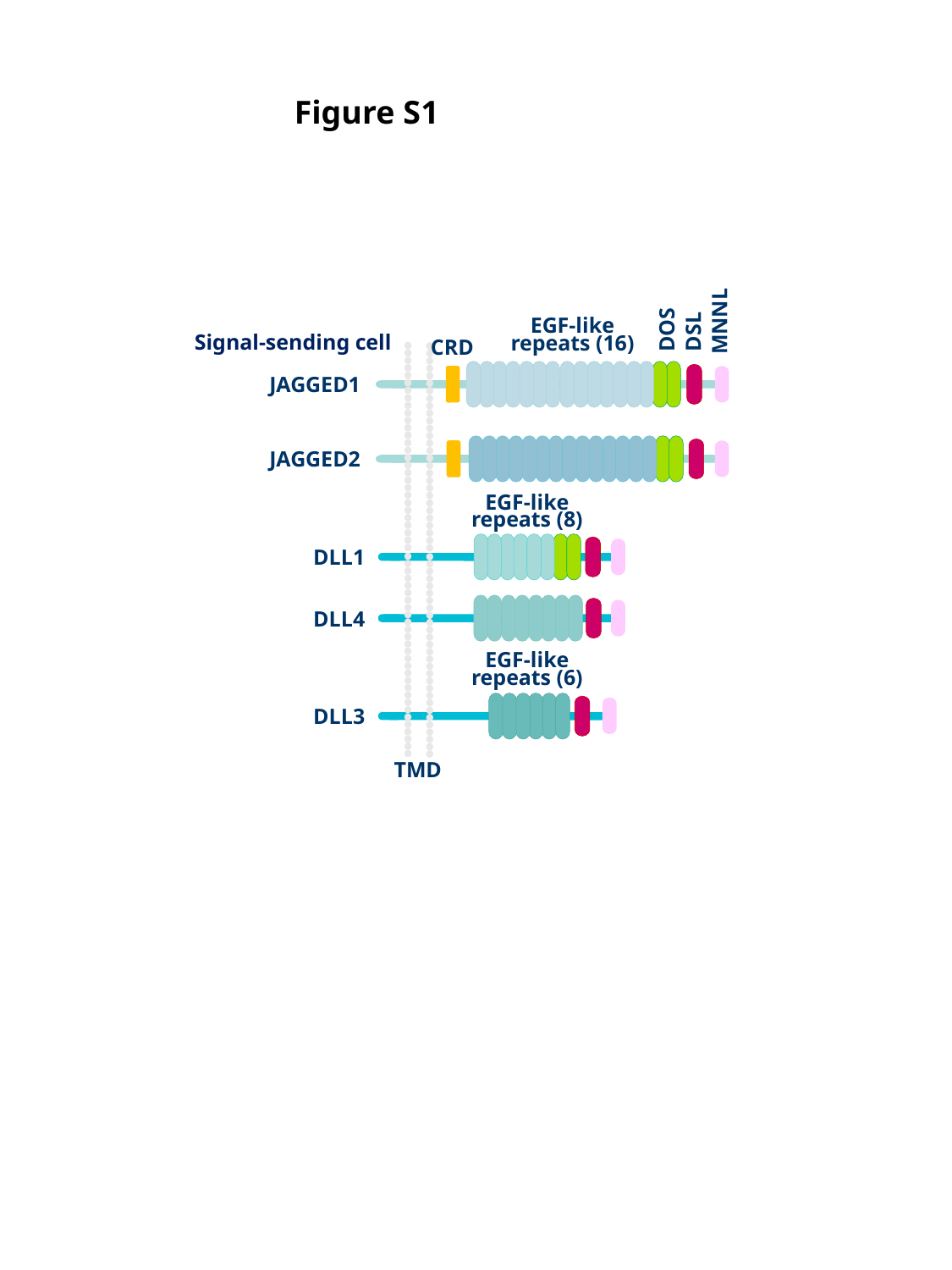

Figure S1
MNNL
DOS
DSL
EGF-like repeats (16)
Signal-sending cell
CRD
JAGGED1
JAGGED2
EGF-like repeats (8)
DLL1
DLL4
EGF-like repeats (6)
DLL3
TMD

## Slide 2
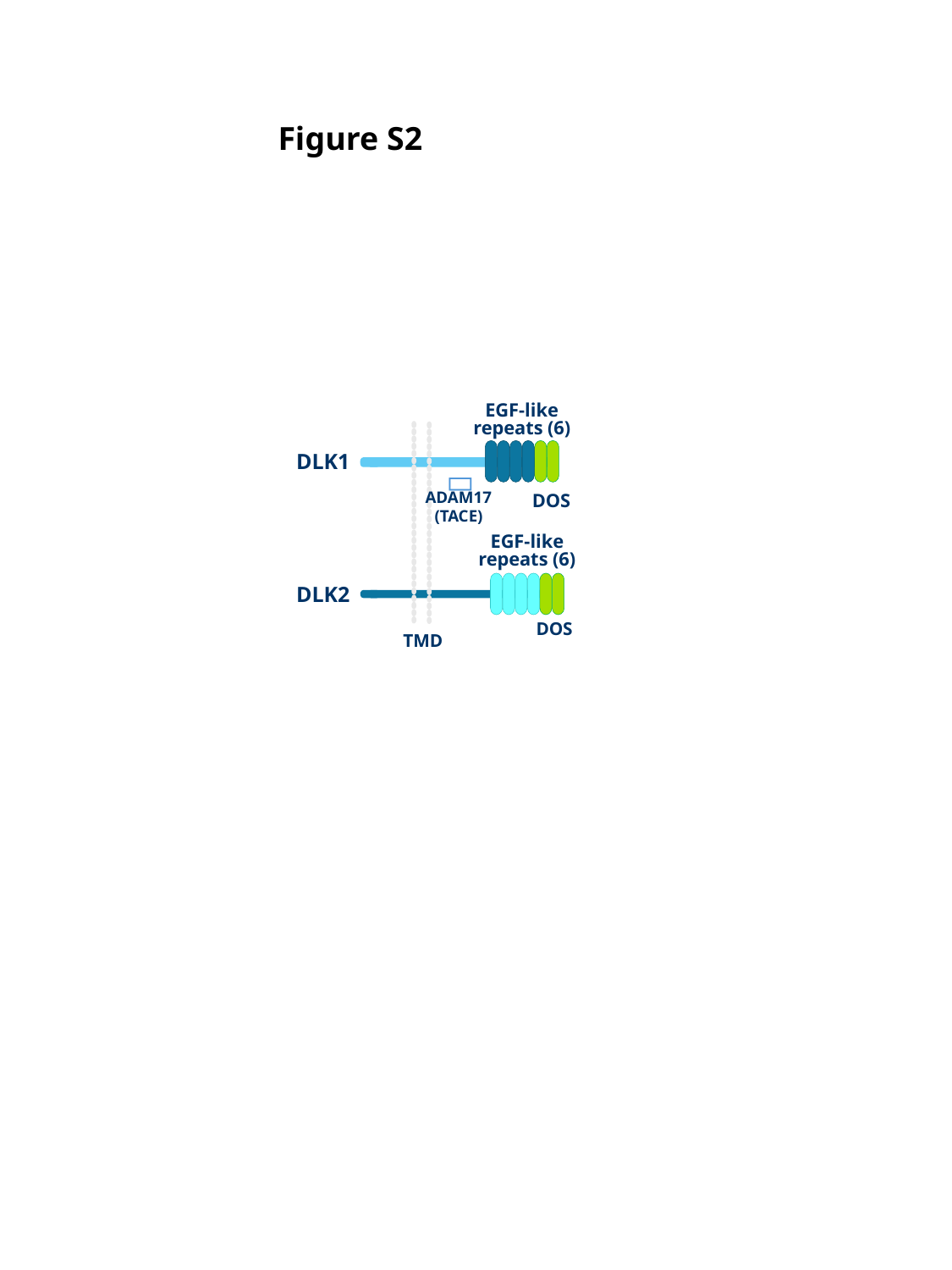

Figure S2
EGF-like repeats (6)
DLK1

ADAM17 (TACE)
DOS
EGF-like repeats (6)
DLK2
DOS
TMD

## Slide 3
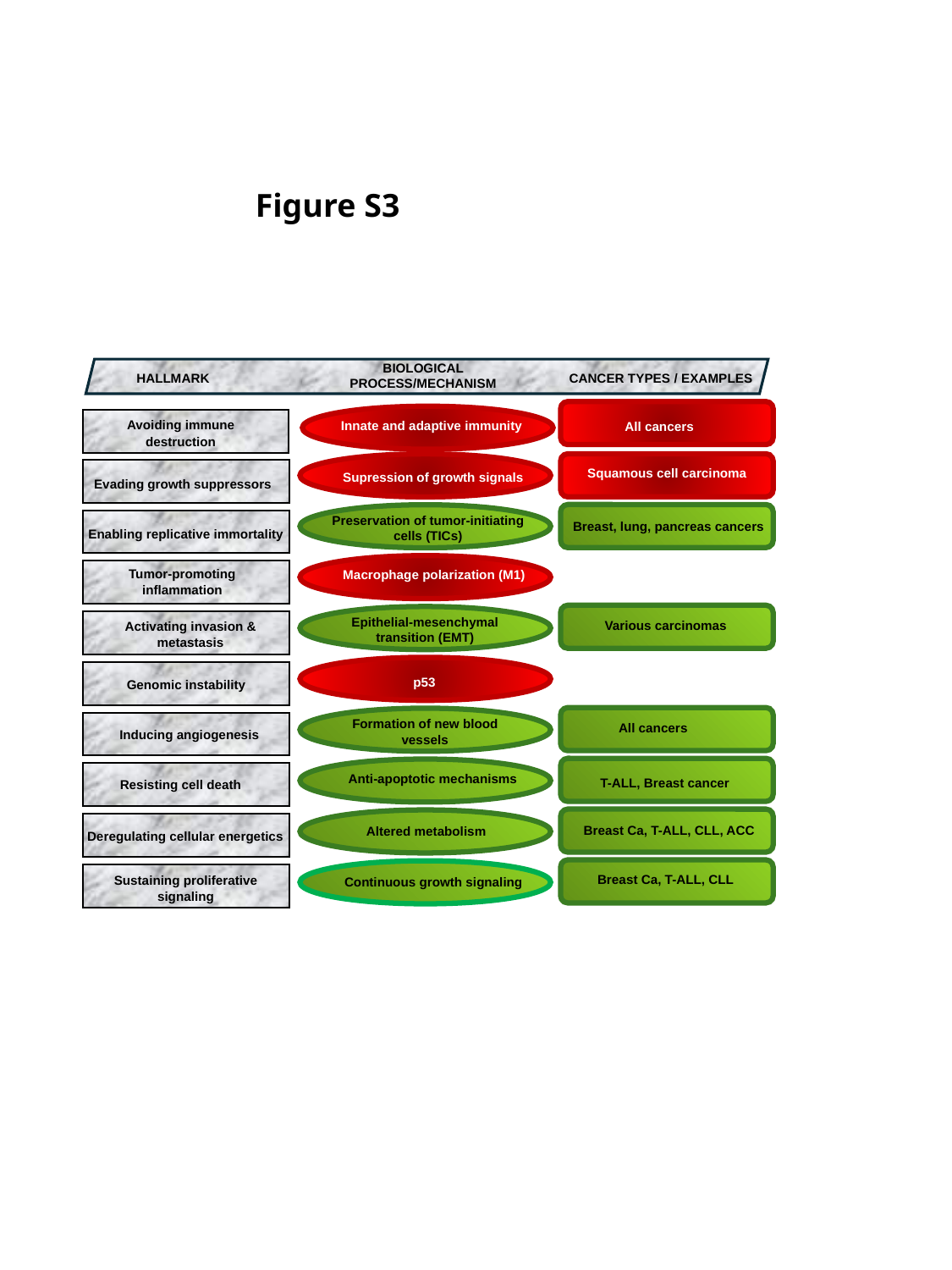

Figure S3
BIOLOGICAL PROCESS/MECHANISM
CANCER TYPES / EXAMPLES
HALLMARK
Avoiding immune destruction
Innate and adaptive immunity
All cancers
Squamous cell carcinoma
Supression of growth signals
Evading growth suppressors
Preservation of tumor-initiating cells (TICs)
Breast, lung, pancreas cancers
Enabling replicative immortality
Tumor-promoting inflammation
Macrophage polarization (M1)
Epithelial-mesenchymal transition (EMT)
Various carcinomas
Activating invasion & metastasis
p53
Genomic instability
Formation of new blood vessels
All cancers
Inducing angiogenesis
Anti-apoptotic mechanisms
T-ALL, Breast cancer
Resisting cell death
Breast Ca, T-ALL, CLL, ACC
Altered metabolism
Deregulating cellular energetics
Sustaining proliferative signaling
Breast Ca, T-ALL, CLL
Continuous growth signaling
